# Human X-linked Intellectual Disability Factor CUL4B Is Required for Post-meiotic Sperm Development and Male Fertility

**DOI:** 10.1038/srep20227

**Published:** 2016-02-02

**Authors:** Chien-Yu Lin, Chun-Yu Chen, Chih-Hsiang Yu, I-Shing Yu, Shu-Rung Lin, June-Tai Wu, Ying-Hung Lin, Pao-Lin Kuo, Jui-Ching Wu, Shu-Wha Lin

**Affiliations:** 1 Department of Clinical Laboratory Sciences and Medical Biotechnology, College of Medicine, National Taiwan University, Taipei, Taiwan; 2 Laboratory Animal Center, College of Medicine, National Taiwan University, Taipei, Taiwan; 3 Department of Bioscience Technology, College of Science, Chung-Yuan Christian University, Taoyuan, Taiwan; 4 Institute of Molecular Medicine, College of Medicine, National Taiwan University, Taipei, Taiwan; 5 Graduate Institute of Basic Medicine, College of Medicine, Fu Jen Catholic University, New Taipei City, Taiwan; 6 Graduate Institute of Basic Medical Sciences, College of Medicine, National Cheng Kung University, Tainan, Taiwan; 7 Department of Obstetrics and Gynecology, National Cheng Kung University Hospital, College of Medicine, National Cheng Kung University, Tainan, Taiwan; 8 Department of Laboratory Medicine, National Taiwan University Hospital, College of Medicine, National Taiwan University, Taipei, Taiwan; 9 Center of Genomic Medicine, National Taiwan University, Taipei, Taiwan

## Abstract

In this study, we demonstrate that an E3-ubiquitin ligase associated with human X-linked intellectual disability, CUL4B, plays a crucial role in post-meiotic sperm development. Initially, *Cul4b*^*Δ*^*/Y* male mice were found to be sterile and exhibited a progressive loss in germ cells, thereby leading to oligoasthenospermia. Adult *Cul4b* mutant epididymides also contained very low numbers of mature spermatozoa, and these spermatazoa exhibited pronounced morphological abnormalities. In post-meiotic spermatids, CUL4B was dynamically expressed and mitosis of spermatogonia and meiosis of spermatocytes both appeared unaffected. However, the spermatids exhibited significantly higher levels of apoptosis during spermiogenesis, particularly during the acrosome phase through the cap phase. Comparative proteomic analyses identified a large-scale shift between wild-type and *Cul4b* mutant testes during early post-meiotic sperm development. Ultrastructural pathology studies further detected aberrant acrosomes in spermatids and nuclear morphology. The protein levels of both canonical and non-canonical histones were also affected in an early spermatid stage in the absence of *Cul4b*. Thus, X-linked CUL4B appears to play a critical role in acrosomal formation, nuclear condensation, and in regulating histone dynamics during haploid male germ cell differentiation in relation to male fertility in mice. Thus, it is possible that CUL4B-selective substrates are required for post-meiotic sperm morphogenesis.

Infertility and sterility represent emerging medical problems that affect modern society, and approximately one out of seven couples suffers from infertility^1^,^2^. Of the latter, half of these cases are associated with the males. To diagnosis male infertility, a reproductive examination is performed along with semen analysis, according to the standards of the World Health Organization^1^. Currently, hormone therapy and assisted reproductive technologies, including *in vitro* fertilization (IVF) and intra-cytoplasmic sperm injections, are used to treat infertility. However, for males with idiopathic infertility, they do not produce qualitatively serviceable sperm, or do not produce sperm at all, according to testicular biopsies. Hence, identification and understanding of the key genetic regulators of the mammalian spermatogenic process is anticipated to effectively improve diagnostic procedures and clinical treatments involving fertility.

Mammalian spermatogenesis is a complex and dynamic process that involves cell division and differentiation in the seminiferous tubules of the testes. Spermatogenesis is subdivided into mitosis and self-renewal of spermatogonia, meiosis of spermatocytes, and differentiation of haploid spermatids^2^,^3^. During the last stage of sperm development, spermiogenesis, haploid spermatids undergo a dramatic morphological transformation to achieve the hydrodynamic shape of mature sperm with condensed nuclei and acrosomes that contain hydrolytic enzymes needed for sperm-oocyte fusion^4^. Acrosomes are formed through polarization of the Golgi vesicle transport system that coalesces into a cap-like structure on top of the condensed nucleus. Nucleus condensation is also a complicated process. A subset of the canonical histones that are associated with chromosomal DNA are replaced by non-canonical histones to facilitate changes in transcriptional activity and the expression of spermiogenesis genes. During spermatid elongation, the chromatin is again remodeled such that the histones are replaced by testis-specific proteins. As a result of these changes, the nucleus becomes condensed and elongated. An intriguing consideration is how post-meiotic spermatids orchestrate these changes in the cytoskeleton, chromatin structure, and vesicular system to undergo such a dramatic transformation.

Only a small percentage of the possible genetic factors related to spermatogenesis and spermiogenesis have been studied in clinical patients and gene-targeted mouse models^5^,^6^. However, the genes that are specifically related to spermatogenesis are highly conserved between mice and humans. Correspondingly, discoveries from mouse model studies may be applicable to human infertility. Several genome-wide studies have detected significant differences between the transcriptional profile of meiotic and post-meiotic spermatogenic germ cells^7^,^8^. Gene ontology analyses have further revealed that the expression of genes involved in protein turnover are elevated. Together, these results indicate that spermiogenesis is highly dynamic and it requires controlled regulation of protein degradation^9^.

E3 ligase proteins in the ubiquitin-proteasome system (UPS) specifically and selectively recognize proteins targeted for ubiquitination. These proteins are hypothesized to play a key role in maintaining functional spermatogenesis. Correspondingly, several E3 ligases have been shown to be crucial for germ cell meiosis^10^,^11^ and spermiogenesis^12^,^13^ in mice, including two members of the cullin protein family, CUL4A and cullin 3. CUL4A together with its homolog, CUL4B, belongs to the CRL4 subfamily, and the CRL4 complex has been shown to play a critical role in the survival of both male and female germ cells. CUL4 utilizes DNA binding protein 1 (DDB1) and DDB1-CUL4 associated factor-1 (DCAF1) as linker protein and substrate receptor respectively to regulate oocyte survival, cumulus expansion and ovulation^14^,^15^. Moreover, CRL4-DCAF1 E3 ubiquitin ligase complex can also control oocyte meiosis. Loss of either DDB1 or DCAF1 result in delayed meiotic resumption and deficiency of meiosis I^16^. Similar function of CRL4 E3 ligase in meiosis progression of spermatocyte had been revealed. Further studies have shown that CUL4A is specifically expressed in meiosis-stage spermatocytes in the testis and is required for proper progression of male meiosis^10^,^17^. In contrast, CUL4B is expressed in spermatogonia but predominantly in post-meiotic spermatids, and barely expressed in spermatocytes. It is hypothesized that CUL4A and CUL4B have distinct functions during spermatogenesis. To date, however the crucial function(s) of CUL4B in the male reproductive system have not been proven.

CUL4B serves as scaffolding proteins for the E3 ubiquitin ligase. Both CUL4A and CUL4B use the same substrate adaptor, DDB1, to target similar substrates for cellular functions, such as regulation of chromosome function^18^,^19^, maintenance of genome integrity^20^ and cell cycle progression^21^. However, it has recently been reported that CUL4B may target certain substrates which play a role in the cellular functions of different organs and specific cells. For example, It is hypothesized that a lack of *Cul4b* in the early development stages will lead to embryonic lethality due to a decrease in transcription factor p21 mRNA in extra-embryonic tissue^22^. Moreover, CUL4B has been shown to regulate neural progenitor cell growth and to target the H3K4 methyltransferase component, WDR5, which modulates neuronal gene expression^23^. Our previous study *of the Cul4b* mutant mouse model^24^, together with several studies of human diseases of X-linked intellectual disability (XLID), have elucidated a role for CUL4B deficiency in the brain^25^,^26^,^27^,^28^. Other CUL4B substrates and adaptor proteins that have been identified include, androgen receptor, which is degraded by CUL4B through its binding of the adaptor protein of dioxin receptor and DDB1^29^, cyclin E^30^, β-catenin^31^, CDT1^32^, MRFAP1^33^, p27^34^, and topoisomerase I^32^. The *in-vivo* significance of the regulation of these interactions by CUL4B has not been intensively studied.

In the present study of the *Cul4b* mutant mice that we have previously generated and characterized to recapitulate the human XLID, it is demonstrated that mammalian CUL4B plays a key role in regulating the early steps of post-meiotic sperm development. Our results enhance the understanding of the molecular basis of CUL4B in spermiogenesis, and they have the potential to facilitate the development of non-hormonal male contraceptives and therapeutic strategies to treat male infertility.

## Results

### CUL4B-deficient male mice are infertile

CUL4B-deficient male (*Cul4b*^*Δ*^*/Y*) mice at P80 were mated with several wild type (WT) females and the number of progeny were recorded. Despite normal sexual behaviors and observable copulatory plugs in the mated females, all 11 *Cul4b*^*Δ*^*/Y* mice failed to sire any progeny within a period of two months. Next, fertility in older (>1-year-old) *Cul4b*^*Δ*^*/Y* males was examined. Of the 10 *Cul4b*^*Δ*^*/Y* mice examined, no progeny were produced. To investigate possible defects in the endocrine system, the anatomical structure, size, and weight ratio of the reproductive organs, as well as serum levels of hormones, between *Cul4b*^*Δ*^*/Y* and WT control mice (*Cul4b*^*+*^*/Y* and *Cul4b*^*lox*^*/Y*) were compared. No significant differences were observed (see Supplementary Fig. S1).

### CUL4B-deficient male mice are defective in sperm production.

**Figure 1:**
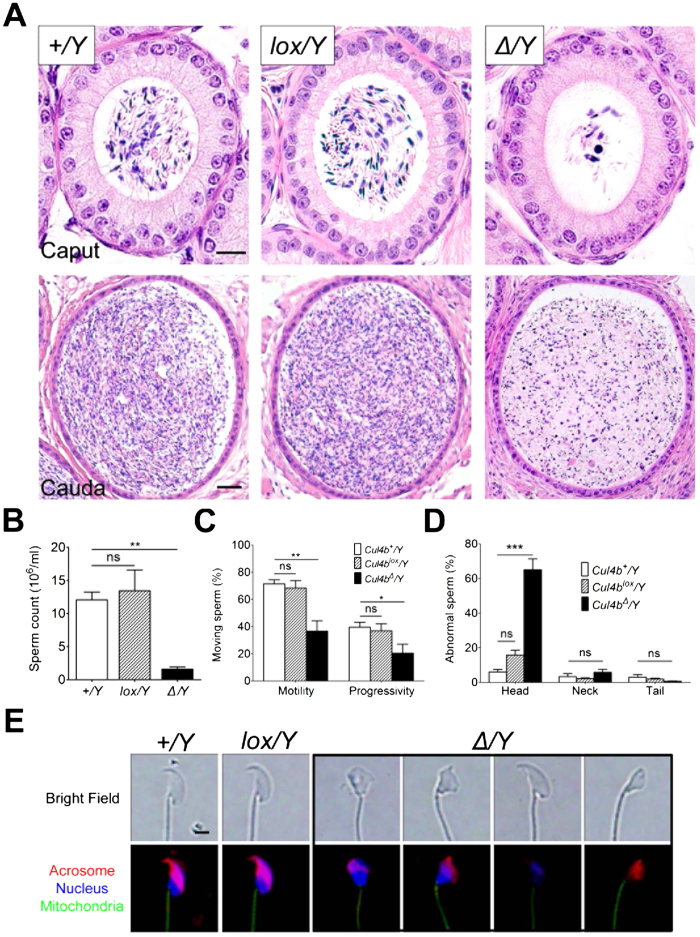
CUL4B-deficient male mice have reduced sperm production and generate defective sperm. (**A**) Representative images from H&E-stained caput and cauda portions of epididymal cross-sections from sexually mature (P80) mice. The amount of spermatozoa within the epididymal lumen of *Cul4b*^*Δ*^*/Y* (^*Δ*^*/Y*) mice was greatly reduced compared to the amount in the *Cul4b*^*+*^*/Y* (*+/Y*) and *Cul4b*^*lox*^*/Y* (*lox/Y*) mice. Scale bars, 10 μm (caput) and 25 μm (cauda). (**B**) Sperm counts within the cauda epididymides for the three genotypes. *Cul4b*^*Δ*^*/Y* mice showed a significant decrease in the number of sperm compared to the other mice (n = 6/group). (**C**) Motility and progressivity were assessed for cauda epididymal sperm from each of the three genotypes. The percentage of isolated free-moving cauda epididymidal sperm exhibiting normal motility and progressivity are reported. Sperm from the *Cul4b*^*Δ*^*/Y* mice exhibited a significant decrease in motility (n = 6/group). (**D**) Quantification of the number of spermatozoa that exhibited abnormalities. Figure S3 shows representative H&E-stained cauda epididymidal spermatozoa that were evaluated for morphological integrity of the head, neck, and tail. *Cul4b*^*Δ*^*/Y* spermatozoa had a significantly higher frequency of spermatozoa with an abnormal head, yet not spermatozoa with an abnormal neck or tail (100 spermatozoa/mouse, n = 5/group). (**E**) Representative fluorescent images of epididymidal spermatozoa. *Cul4b*^*Δ*^*/Y* sperm exhibited various hookless heads, as well as club-shaped heads, amorphous heads, disintegrated nuclei, and no nuclei. Scale bar, 2 μm. All values are presented as the mean ± SEM. ANOVA test; ^*^P < 0.05; ^**^P < 0.01; ^***^P < 0.001; ns, not significant).

A histological examination of the *Cul4b*^*Δ*^*/Y* epididymis revealed a dramatic reduction in the amount of sperm produced in both the proximal (caput) and distal (cauda) parts of the epididymis compared to WT mice (Fig. 1A and see Supplementary Fig. S2 online). Thus, a major loss of sperm cells in CUL4B-deficient mice occurs prior to their release from the testes. Next, sperm were isolated from the cauda epididymis, which contains most of the mature sperm cells. A marked reduction in sperm count (Fig. 1B), and decreased sperm motility and progressivity (Fig. 1C and see Supplementary Videos S1), were associated with the *Cul4b*^*Δ*^*/Y* (^*Δ*^*/Y*) and *Cul4b*^*Δ*^*/Y* male mice compared to the WT male mice. Furthermore, up to 65% of the isolated *Cul4b*^*Δ*^*/Y* sperm exhibited morphologically defective heads, compared to 6–16% of WT sperm (Fig. 1D, and see Supplementary Fig. S3).

Using immunofluorescence staining, various hookless heads, including those with club-shaped heads, amorphous heads, disintegrated nuclei, and no nuclei, were observed among the *Cul4b*^*Δ*^*/Y* sperm (Fig. 1E). Correspondingly, when *in vitro* fertilization (IVF) experiments were performed with adjusted sperm concentrations, ~4-times fewer fertilization events were recorded for *Cul4b*^*Δ*^*/Y* sperm compared to WT sperm (see Supplementary Fig. S4 online). Thus, *Cul4b*^*Δ*^*/Y* male mice produce fewer and more defective sperm that are primarily culled prior to exit from the testes.

### Dynamic localization of CUL4B during post-meiotic germ cell development

**Figure 2:**
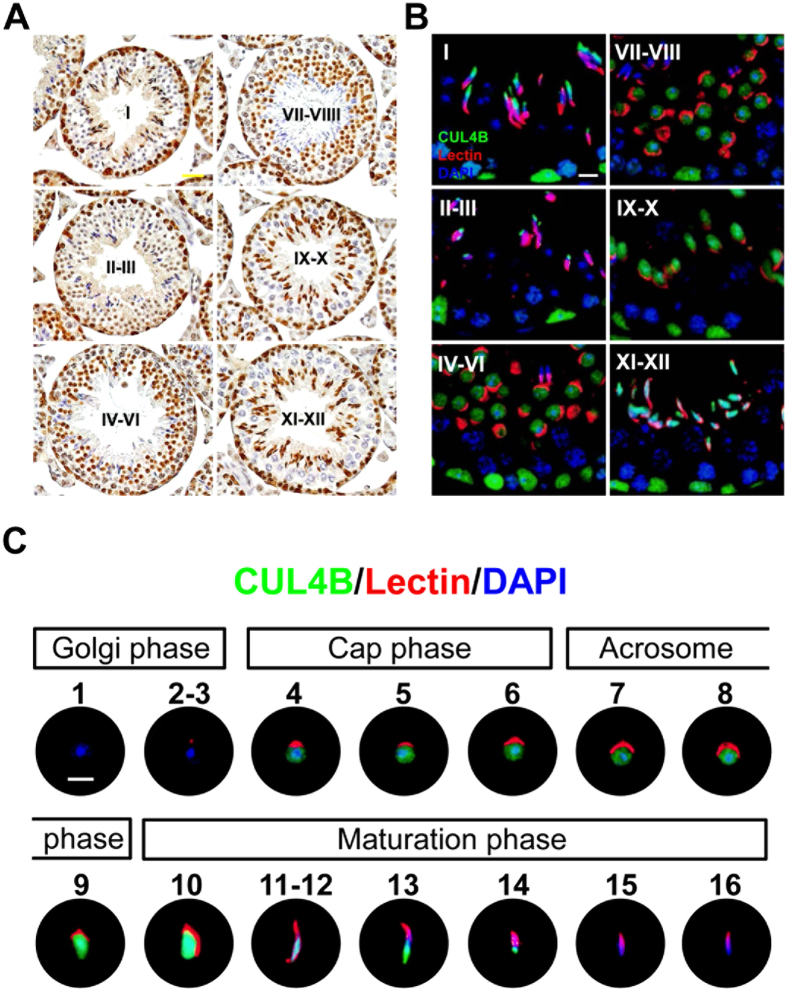
Stage-specific localization of CUL4B during spermatogenesis. (**A**) Immunohistochemical staining for CUL4B expression (nuclear brown staining) in testicular sections prepared from mature WT mice. The sections were counterstained with hematoxylin to help differentiate between the twelve maturation stages (I–XII) of the seminiferous tubules. (**B**) Enlarged regions of the immunofluorescent stainings performed to detect CUL4B (red), the acrosome marker, lectin (green), and chromatin (DAPI, blue) for the different maturation stages indicated. CUL4B was persistently expressed throughout spermatogonia, was dynamically expressed in spermatids, and was not detected in spermatocytes. (**C**) Spermatids from adult mice that are representative of the 16 progressive steps in spermiogenesis observed in six independent experiments. CUL4B was first detected in the step 4 round spermatids, it reached its highest expression in the step 10 elongating spermatids, and then disappeared in the elongated spermatids after step 14. Scale bars, 20 μm (**A**), 10 μm (**B**) and 5 μm (**C**).

To elucidate the function of CUL4B in sperm development, immunohistochemical staining of CUL4B was performed for testes tissue sections (Fig. 2A). In adult (P80) WT testes, CUL4B was expressed only in the nuclei of specific cell types including: the early germ cells at the basement of the seminiferous epithelium of all stages of the tubules’ cycles; the round spermatids in the stage IV–VIII tubules; the elongating stage IX–XII spermatids; and the elongated spermatids in stage I tubules (Fig. 2A). In contrast, no discernible CUL4B signals were observed in the corresponding *Cul4b*^*Δ*^*/Y* samples, thereby confirming the loss of CUL4B expression in the testes (data not shown).

Immunofluorescence staining was also performed for testis sections staged with various developing post-meiotic germ cells (Fig. 2B,C), with lectin co-staining used to indicate the developmental steps of the spermatids. Initially, Golgi phase germ cells were absent (steps 1–3), then CUL4B was detected at the beginning of the cap phase (step 4) (Fig. 2C). From the cap phase to the acrosome phase (steps 4–8), CUL4B was diffusely distributed throughout the nucleus. At the transition between the acrosome phase and the maturation phase (steps 9–10), the intensity of CUL4B staining increased abruptly, and was highly associated with the entire condensing nuclei. During the maturation phase, CUL4B expression gradually retreated away from the apical region of the condensing nuclei (steps 11–14), and eventually disappeared in the elongating spermatids (steps 15–16). Notably, CUL4B never co-localized with the developing acrosomes (Fig. 2B,C).

### Loss of *Cul4b* leads to fewer post meiotic spermatids in the adult spermatogenic cycle

To evaluate post-meiotic sperm development, histological analyses were performed. *Cul4b*^*Δ*^*/Y* tubules were significantly void of mature sperm compared to WT tubules, suggesting the former were hypocellular for germ cells. Nonetheless, the number of seminiferous tubule per section and the diameter of the tubules were comparable between *Cul4b*^*Δ*^*/Y* mice and WT mice (see Supplementary Fig. S5 online). Using immunohistochemical staining, the number of Sertoli cells and spermatocytes were comparable between *Cul4b*^*Δ*^*/Y* and WT mice (see Supplementary Fig. S6 online). Thus, loss of sperm appears to occur post-meiotically in *Cul4b*^*Δ*^*/Y* tubules.

**Figure 3:**
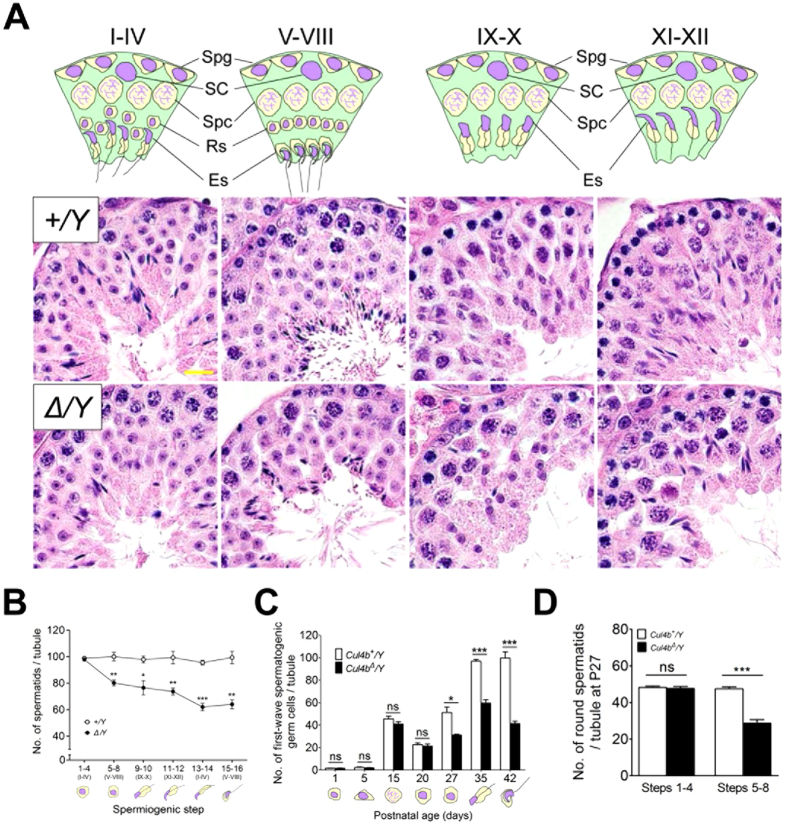
Stage-specific loss of post-meiotic spermatids in CUL4B-deficient testes. (**A**) H&E-stained testicular sections from adult *Cul4b*^*+*^*/Y* (*+/Y*) and *Cul4b*^*Δ*^*/Y* (^*Δ*^*/Y*) mice. The spermatogenic stages were divided into four groups according to the presence of specific cell types and their arrangements: I–IV, V–VIII, IX–X, and XI–XII. Fewer spermatids were present in the seminiferous tubules of the *Cul4b*^*Δ*^*/Y* mice compared with the *Cul4b*^*+*^*/Y* mice through all four stages. Scale bar, 10 μm. (**B**) The number of post-meiotic spermatids counted for the specific steps in H&E-stained adult testicular sections. A gradual decrease in steps 5–16 spermatids was observed in the *Cul4b*^*Δ*^*/Y* sections. (**C**) The number of developing germ cells present during the first wave of spermatogenesis. Gonocytes, spermatogonia, spermatocytes, and spermatids were counted from H&E-stained testicular sections at the postnatal days indicated. After meiosis, a significant reduction in spermatid counts was observed from P27–P42 for the *Cul4b*^*Δ*^*/Y* mice. (**D**) The number of steps 1–4 and 5–8 spermatids counted from H&E-stained testicular sections at P27. A decrease in the total number of spermatids in *Cul4b*^*Δ*^*/Y* mice at P27 is attributed to a loss of steps 5–8 spermatids. For B–D, 20 tubules were counted for each stage/mouse, n = 6/group. All values are presented as the mean ± SEM. Student’s *t*-test; ^*^P < 0.05; ^**^P < 0.01; ^***^P < 0.001; ns, not significant.

The number of post-meiotic spermatids at each step of spermiogenesis were also examined in microscopic sections. In stage I–IV tubule sections, comparable numbers of round spermatids were observed between *Cul4b*^*Δ*^*/Y* and WT mice (Fig. 3A,B, and see Supplementary Table S2 online). However, in *Cul4b*^*Δ*^*/Y* stage V–VIII tubules, a 20% decrease in round spermatids was observed in steps 5–8 compared to the corresponding WT tubules (Fig. 3A,B, and see Supplementary Table S2 online). In the stage IX–X and XI–XII tubules, which contain steps 9–12 elongating spermatids, a cumulative decrease in spermatids was observed (Fig. 3A,B and see Supplementary table S2 online). Finally, in steps 13–14 and 15–16, very few elongated spermatids were observed in the stage I–IV and V–VIII tubules of the *Cul4b*^*Δ*^*/Y* mice, while the WT testes contained large numbers of late-stage spermatids (Fig. 3A,B, and see Supplementary Table S2 online). These results suggest that developing sperm are progressively removed from *Cul4b*^*Δ*^*/Y* tubules, starting at the transition from the Golgi phase (steps 1–4) to the cap phase (steps 5–8).

### Loss of *Cul4b* leads to fewer haploid spermatids in the first wave spermatogenesis

To pinpoint the exact spermiogenesis step(s) that are CUL4B-dependent, testes were isolated from male mice at different postnatal ages. Using SCP3 as a meiosis marker, the progression of meiotic entry in the first wave spermatogenesis was observed (Table 1). In both WT and *Cul4b*^*Δ*^*/Y* mice, more than 80% of the tubules were positive for meiosis by P15, indicating that normal meiosis entry occurs in *Cul4b*^*Δ*^*/Y* mice. Correspondingly, H&E staining showed the number of germ cells present in the first wave spermatogenesis of *Cul4b*^*Δ*^*/Y* tubules were comparable to WT tubules up to P20 (Fig. 3C). Thus, spermatogonia division and spermatocyte meiosis were unaffected (see Supplementary Fig. S6 online). Next, progression of post-meiotic entry was examined. Both WT and *Cul4b*^*Δ*^*/Y* mice were positive for the acrosome marker, PNA lectin (Table 1), and thus, progression of post-meiotic entry was found to be unaffected in *Cul4b*^*Δ*^*/Y* mice.

Despite exhibiting normal meiotic and post-meiotic entry, *Cul4b*^*Δ*^*/Y* mice showed a progressive decrease in germ cell number after P27 (Fig. 3C). P27 contains both steps 1–4 (Golgi to cap phase transition) and steps 5–8 (cap to acrosome phase transition) round spermatids. Therefore, the number of these two cell groups were counted in P27 tubules from WT and *Cul4b*^*Δ*^*/Y* tubules. While the number of steps 1–4 round spermatids were unaffected, the number of steps 5–8 round spermatids declined by ~50% in the *Cul4b*^*Δ*^*/Y* tubules (Fig. 3D). Thus, in *Cul4b*^*Δ*^*/Y* mice, significant round spermatid loss occurs during the transition from the cap phase to the acrosome phase during spermiogenesis.

### Loss of CUL4B leads to increased sperm apoptosis during the cap phase and acrosome phase

**Figure 4:**
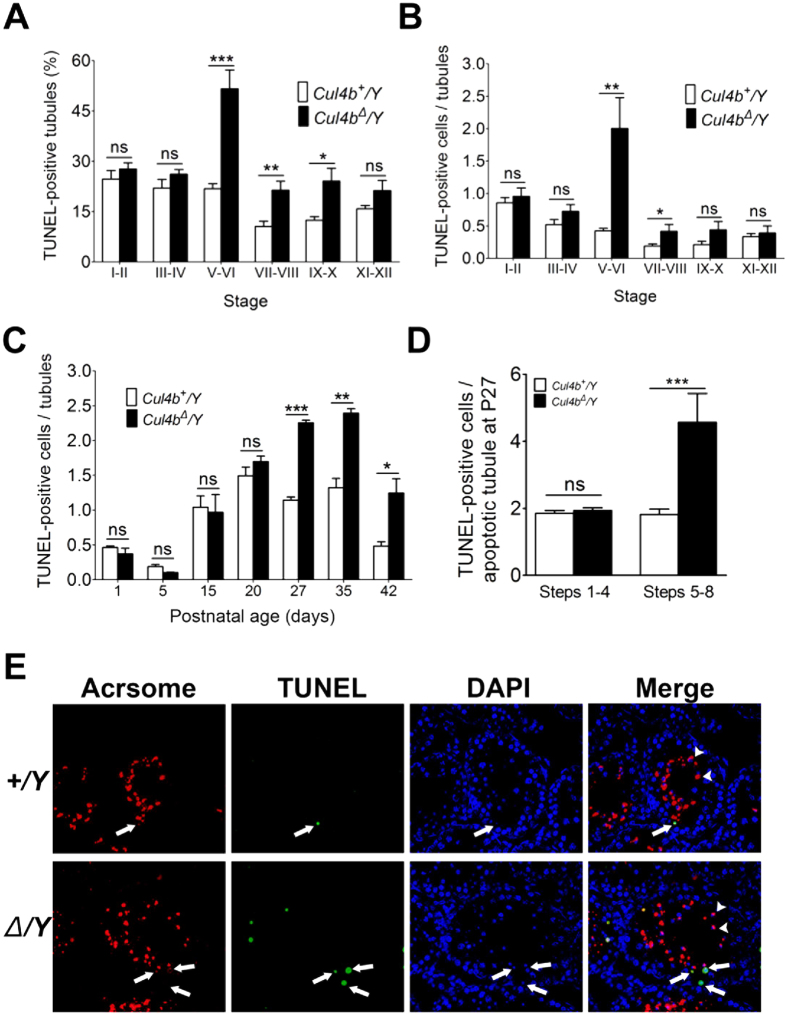
Loss of CUL4B results in elevated cell death in steps 5–8 spermatids. (**A,B**) Quantitative TUNEL analysis of adult mice testes sections. The percentage of TUNEL-positive tubules (**A**) and the number of TUNEL-positive cells (**B**) detected at different spermatid stages (100 tubules/mouse, n = 6/group). (**C–E**) Quantitative TUNEL analysis during the first wave of spermatogenesis. (**C**) TUNEL-positive cells were counted for each of the sections collected at the postnatal days indicated. (**D**) The distribution of TUNEL-positive spermatids between steps 1–4 and 5–8 at P27. (20 tubules per postnatal day/mouse, n = 6/postnatal group). All values are presented as the mean ± SEM. Student’s *t*-test; ^*^P < 0.05; ^**^P < 0.01; ^***^P < 0.001; ns, not significant. (**E**) Representative images of P27 *Cul4b*^*+*^*/Y* and *Cul4b*^*Δ*^*/Y* testicular sections co-stained with TUNEL (green), lectin (red), and DAPI (blue). Co-localization of TUNEL and lectin signals suggested that a greater of apoptosis events were observed in tubules with steps 5–8 spermatids in *Cul4b*^*Δ*^*/Y* mice at P27. Arrows indicate TUNEL-positive cells and arrowheads indicate steps 5–8 spermatids. Scale bar, 20 μm.

To examine whether the decreased number of spermatids in *Cul4b*^*Δ*^*/Y* testes was due to apoptosis, TUNEL analyses were performed (Fig. 4 and see Supplementary Fig. S7 online). Both TUNEL-positive tubules and cells were significantly increased in P80 *Cul4b*^*Δ*^*/Y* whole testicular sections compared with WT sections (see Supplementary Fig. S7 online). Furthermore, apoptosis occurred in tubules at all stages in the WT testes (Fig. 4A,B), with 20–25% of stage I–II, III–IV, and V–VI tubules being TUNEL-positive, and ~10% of stage VII–VIII and IX–X tubules being positive. However, in *Cul4b*^*Δ*^*/Y* testes, a significant increase in the numbers of TUNEL-positive tubules were observed from stages V–VIII (Fig. 4A), with ~50% of stage V–VI tubules being TUNEL-positive (representing a two-fold increase compared to WT tubules at the same stage) (Fig. 4A). Stage V–VI *Cul4b*^*Δ*^*/Y* tubules also showed a four-fold increase in the number of apoptotic cells per tubule compared with the corresponding WT tubules (Fig. 4B). When stage-specific apoptosis was examined up to stage P20, both WT and *Cul4b*^*Δ*^*/Y* tubules showed comparable numbers of apoptotic germ cells (Fig. 4C and see Supplementary Fig. S8 online). Nonetheless, from P27 on, a significant increase in the number of apoptotic cells in the *Cul4b*^*Δ*^*/Y* tubules were observed (Fig. 4C and see Supplementary Fig. S8 online). To examine whether specific groups of germ cells are involved (Fig. see Supplementary Fig. S9 online), immunofluorescence staining for apoptosis and the acrosome marker, PNA lectin, were performed (Fig. 4E). P27 *Cul4b*^*Δ*^*/Y* tubules contained more than twice the number of apoptotic steps 5–8 tubules (see Supplementary Fig. S9 online) and spermatids (Fig. 4D) compared to WT, while the frequency and number of apoptotic steps 1–4 tubules and spermatids were comparable (Figs. 4D). Therefore, in *Cul4b*^*Δ*^*/Y* mice, increased apoptosis occurs in steps 5–8 spermatids, and this eventually leads to a loss of sperm cells in the later stages.

### CUL4B modulates protein factors that are required for post-meiotic sperm morphology transformation

**Figure 5:**
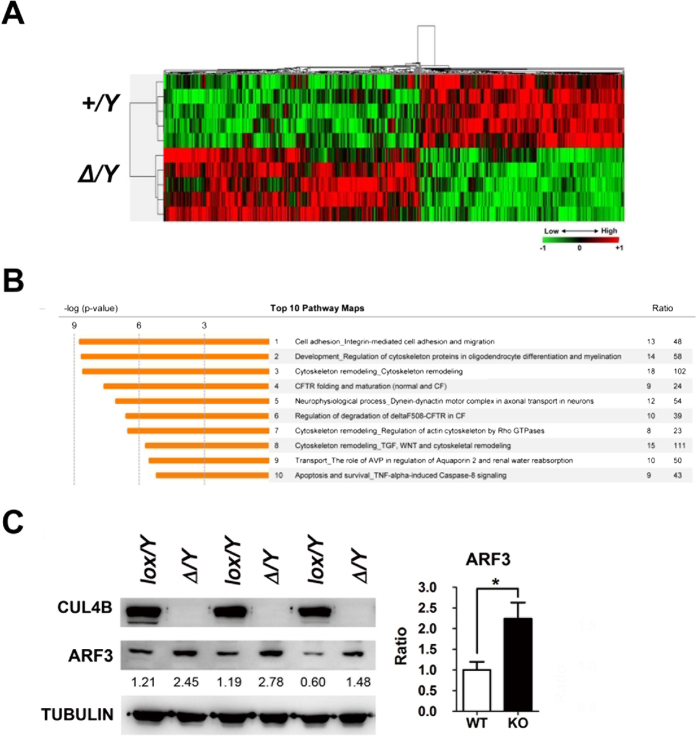
Functional categorization of the differentially expressed proteins identified between wild-type and CUL4B-deficient testes tissues collected at P20. (**A**) Heat map analysis and hierarchical clustering represent the proteomic profiles of differentially expressed proteins in testes tissues collected from *Cul4b*^*+*^*/Y* (*+/Y*) and *Cul4b*^*Δ*^*/Y* (^*Δ*^*/Y*) mice (*P* < 0.05). Green indicates reduced expression levels and red indicates increased expression levels relative to *+/Y*. (n = 5/group). (**B**) Functional ontologies associated with the proteomic data are presented as a list of the top ten pathways identified by using MetaCore analysis software and predicted potential functions of CUL4B in post-meiotic spermatogenesis. (**C**) Up-regulated protein level of ARF3 in proteomic analysis was confirmed using immunoblooting. Detection of tubulin was used as a loading control. Densitometry values are indicated with the levels of ARF3 and the average signal of the *lox/Y* extract is set to 1. ^*^P < 0.05.

Our results indicate that CUL4B may play an important role in early stage spermatids following completion of meiosis. As an E3-ubiquitin ligase, CUL4B has been reported to modulate various cellular processes by regulating the degradation of specific cellular factors^20^. We have analyzed the levels of proteins that have been identified as CUL4B substrates and adaptor proteins, including, DDB1, androgen receptor, β-catenin, CDT1, cyclin E, MRFAP1, p27, topoisomerase I, and WDR5, and did not find significant changes in their expression levels in testes of adult *Cul4b*^*Δ*^*/Y* mice compared to those of WT mice (data not shown). Therefore, we decided to perform proteomics and compare collect protein samples from WT and *Cul4b*^*Δ*^*/Y* testes at P20, when CUL4B is first detected in WT testes and massive spermatid loss has not taken place in *Cul4b*^*Δ*^*/Y*. The samples were trypsinized and underwent liquid chromatography-tandem mass spectrometry (LC-MS/MS) as quintuplicate samples. Of the 2,095 unique proteins that were identified, 685 were differentially expressed between WT and *Cul4b*^*Δ*^*/Y* testes samples (Fig. 5A, and see Supplementary File S1). Functional ontology analyses of the latter identified four pathways related to cytoskeletal remodeling, and another four pathways related to intracellular transportation/sorting (Fig. 5B). These results are consistent with a proposed role for CUL4B in rearranging structural factors required for subcellular organelle transformation in spermatids upon completion of meiosis. To confirm the results from proteomic analyses, we examined the levels of ADP-ribosylation factor 3 (*Arf3*), a vesicular trafficking regulator revealed to be upregulated in *Cul4b*^*Δ*^*/Y* mice testes. According to the immunoblotting data, ARF3 protein of *Cul4b*^*Δ*^*/Y* mice testes demonstrates definitely upregulation compared to WT mice testes (Fig. 5C). This result provides the evidence of reliability of comparative proteomic analysis.

### CUL4B is required for proper acrosome formation and nuclear morphogenesis during spermiogenesis

**Figure 6:**
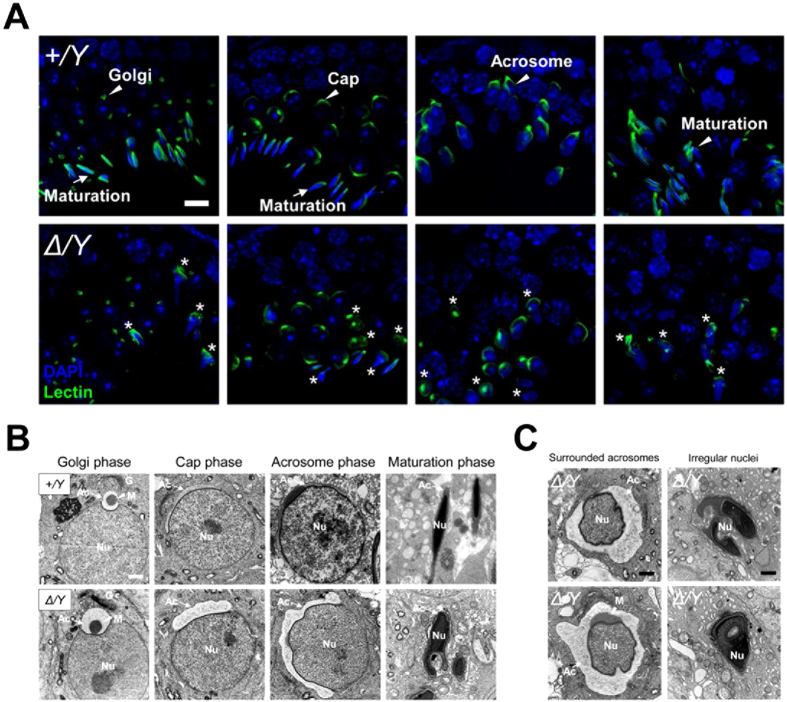
Defective acrosome formation and nuclear condensation were detected in CUL4B-deficient spermatids. (**A**) Confocal images of adult *Cul4b*^*+*^*/Y* and *Cul4b*^*Δ*^*/Y* testicular sections co-stained with the acrosome marker, lectin (green), and DAPI (blue). Regions containing growing acrosomes are indicated as Golgi, cap, acrosome, and maturation phases, and these were accompanied by round, elongating, and elongated nuclei in spermatids during spermiogenesis. Arrowheads and arrows indicate normally developing acrosomes and condensed nuclei in *Cul4b*^*+*^*/Y* spermatids. Asterisks indicate aberrant acrosomes and irregular nuclei in *Cul4b*^*Δ*^*/Y* spermatids. (**B**) TEM micrographs of spermatids in four progressive phases of spermiogenesis. In *Cul4b*^*+*^*/Y* spermatids, the acrosomal vesicle remained rounded on the nuclear surface and the vesicle flattened and covered the head of the nucleus in the cap phase. By the maturation phase, sperm chromatin had condensed to fit in a pencil-shaped nuclei. In *Cul4b*^*Δ*^*/Y* spermatids, the acrosomal vesicles were loose and the nuclei were irregular. (**C**) TEM micrographs show *Cul4b*^*Δ*^*/Y* spermatids with surrounding acrosome structures and irregularly shaped nuclei. Abbreviations: G, Golgi apparatus; Nu, nucleus; Ac, acrosome; M, acrosomal matrix. Scale bars, 5 μm (**A**) and 1 μm (**B,C**). All values represent the mean ± SEM. Student’s *t*-test; ^*^P < 0.05; ns, not significant.

Based on the proteomic results obtained, acrosome formation in *Cul4b*^*Δ*^*/Y* mice was examined at different spermiogenesis stages. The development of the acrosome can be visualized by staining with fluorescently-labeled lectin (Fig. 6A). In *Cul4b*^*Δ*^*/Y* testes, spermatids with normal Golgi phase acrosomes were detected. However, while the acrosome appeared to be maintained as a heavily labeled dot with thin extensions over the nucleus (Fig. 6A), various acrosome abnormalities were observed, including under-extension of the acrosome and over-extension of the acrosome to encircle the entire nuclei. Furthermore, in sections exhibiting normal maturation of the acrosome, most spermatids were lost, while the few remaining spermatids exhibited an abnormal morphology (Fig. 6A). These results indicate that acrosome formation is disrupted in *Cul4b*^*Δ*^*/Y* testes.

Developing spermatids were further examined with transmission electron microscopy (Fig. 6B,C). In *Cul4b*^*Δ*^*/Y* mice, the ultrastructure of the acrosomes and the matrix were similar to those of WT mice during the Golgi phase. However, in the subsequent stages (Fig. 6B), instead of the acrosomes being smoothly flattened and extended to cover one-third of the nucleus, developing acrosomes of the *Cul4b*^*Δ*^*/Y* mice often failed to be smoothened and flattened, thereby leaving an irregularly shaped vacuole-like structure surrounding the nuclei (Fig. 6B,C). In addition to abnormal acrosome morphogenesis, the nuclei of the developing spermatids were characterized by patchy and heterogenous domains that were undergoing reorganization (Fig. 6B). The shape of the nuclei of many of the CUL4B-deficient developing spermatids was also irregular with ruffles (Fig. 6B,C). As a result, smoothly condensed rod-shaped nuclei were difficult to observe during the maturation phase in *Cul4b*^*Δ*^*/Y* mice. Thus, both acrosome formation and nuclear morphogenesis are defective in *Cul4b*^*Δ*^*/Y* mice.

### CUL4B regulates histone turnover in post-meiotic spermatozoa

**Figure 7:**
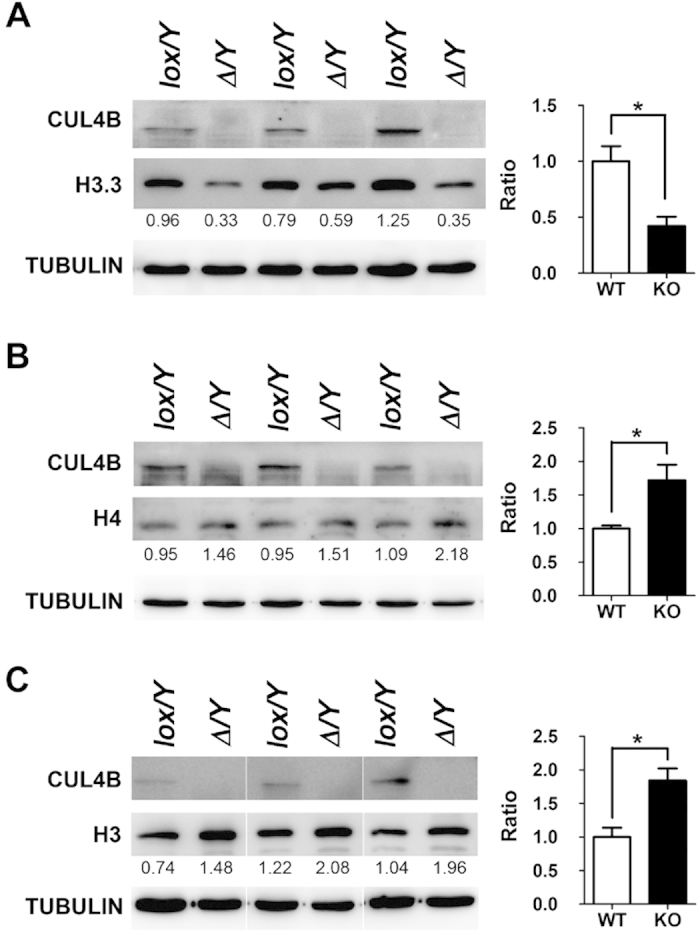
Immunoblotting to detect protein levels of canonical and variant histones in wild-type and CUL4B-deficient testes at P20. (**A**) Lower levels of testis-specific histone H3.3, higher expression levels of (**B**) histones H4 and (**C**) H3, and, were detected in *Cul4b*^*∆/*^*Y* (^*Δ*^*/Y*) testes extracts compared to *Cul4b*^*lox/*^*Y* (*lox/Y*) testes extracts. Detection of tubulin was used as a loading control. Densitometry values are indicated with the levels of histones H3.3, H4, and H3 and the average signal of the *lox/Y* extract is set to 1.

In previous studies, CUL4B has been shown to participate in various posttranslational modifications of histones^35^,^36^. Therefore, expression levels of canonical histones and the testis-specific histone, H3.3, were compared between WT and *Cul4b*^*Δ*^*/Y* testes at stage P15 and P20. Consistent with the results from our proteomic analyses, levels of histone H3 and histone H4 were found to be elevated in P20-stage *Cul4b*^*Δ*^*/Y* testes compared to WT testes, while significantly lower levels of histone H3.3 were detect in *Cul4b*^*Δ*^*/Y* testes (Fig. 7). However protein levels of histone H3.3, H3 and histone H4 showed no different at P15-stage testes, which have no post-meiotic cells, compared between WT and *Cul4b*^*Δ*^*/Y* testes (see Supplementary Fig. S10 online). These results suggest that CUL4B is required for post-meiotic sperm histone turnover, and this affects chromatin remodeling.

## Discussion

In this study, we present evidence that the dynamic expression of mammalian CUL4B in post-meiotic spermatids is a key factor in regulating the early steps of post-meiotic sperm development. Moreover, in the absence of CUL4B, sperm production is greatly reduced due to elevated apoptosis in spermatids undergoing spermiogenesis. A proteomic analysis further revealed changes in the expression of factors that mediate cytoskeletal remodeling and intracellular transportation pathways, both of which are required for morphological changes. In our immunofluorescence and electron microscopy analyses, aberrant acrosome structures were observed and both the vesicular and chromatin remodeling systems are misregulated post-meiotically in the absence of CUL4B, the latter of which affected the turnover of canonical histones and the incorporation of testis-specific histones. Thus, CUL4B appears to be crucial for regulating a genome-wide coordination of the transformation that occurs during spermiogenesis.

### Collaborative roles of CUL4B and CUL4A in sperm development

CUL4B is highly related to CUL4A, and these two proteins have been implicated in redundant or complementary roles involving protein turnover^20^,^37^. Previous studies have revealed that loss of DDB1 which associates with both CUL4A and CUL4B causes defect in oocyte survival, oocyte meiotic maturation and post-meiotic oocyte maintenance^14^,^15^,^16^. This suggests that CRL4 E3 ubiquitin complex plays essential role in germ cell development. In agreement with speculation, both CUL4A- and CUL4B-deficient mice are male sterile^10^,^38^. Since CUL4A and CUL4B display complementary expression pattern during spermatogenesis, it appears that the two cullins mediate non-compensatory effects during sperm production. CUL4A is primarily expressed in the cytoplasm of spermatocytes during meiosis, while CUL4B is mainly expressed in the nuclei of spermatogonia during mitosis and in haploid post-meiotic spermatids^38^. In combination with the present data, CUL4A appears to be required for sperm meiosis, while CUL4B is required for post-meiotic sperm development. Despite CUL4B, and not CUL4A, is expressed in spermatogonia, However significant defects in mitosis and in the differentiation of CUL4B-deficient spermatogonia were not observed. Thus, the role of CUL4B in spermatogonia remains unclear.

Two possible schemes would account for the different expression pattern of CUL4A and CUL4B during spermatogenesis. First, the transcription of *Cul4b* mRNA begins in spermatocyte but is not translated until spermatid. This gene expression pattern is similar to protamine mRNA which is expressed in spermatocyte but translated after meiosis^39^. Alternatively, the uncommon expression pattern of CUL4B could due to meiotic sex chromosome inactivation. In both humans and mice, *CUL4B* (*Cul4b*) is located on the X chromosome, while *CUL4A* (*Cul4a*) is located on autosomes. In mammals, sex chromosomes generally undergo meiotic sex chromosome inactivation^40^, although several studies have shown that X-lined genes can be expressed post-meiotically^41^. After meiosis, haploid spermatids are generated from the same spermatocytes and they share gene transcripts and proteins through intercellular cytoplasmic bridges (or ring canals)^41^. Therefore, spermatids harboring X chromosomes produce *Cul4b* transcripts and CUL4B proteins, and then share these with neighboring Y chromosomal-bearing gametes via an intercellular trafficking system. Correspondingly, the present results indicate that CUL4B is present in undifferentiated spermatogonia, it is silenced in spermatocytes, and then it reappears in round spermatids due to post-meiotic reactivation of X chromosome transcription. This expression pattern for CUL4B is complementary to that of CUL4A, which is only expressed in meiosis-stage spermatocytes^38^. Therefore, it appears that CUL4B and CUL4A have collaborative roles in regulating proper sperm production throughout spermatogenesis.

Male patients with XLID often display hypogonadism in addition to intellectual disabilities^25^,^26^, and several studies have related this hypogonadism with hormonal deficiencies. However, it remains unclear whether the progression of spermatogenesis is also defective in males with XLID. The present finding that CUL4B is required specifically for post-meiotic spermiogenesis in mice suggests that sperm development may also be affected in males with XLID. It will be important for future studies to investigate this possibility, in addition to the role of hormonal deficiencies, in the observed hypogonadism of males with XLID.

### CUL4B as a key regulator of post-meiotic sperm morphogenesis

Post-meiotic spermiogenesis consists of a series of rearrangements among various cellular components in order to establish morphological changes that are critical for sperm function. For example, the secretory system is remodeled into a specialized acrosome which houses enzymes that facilitate the penetration and fusion of a sperm with an egg^2^,^42^. Meanwhile, spermatogenic chromatin undergoes drastic remodeling, with the sequential replacement of canonical histones with transition proteins and then protamines, to achieve a highly compacted state^43^. Therefore, the results of the proteome comparison that was made between unlabeled WT and *Cul4b*^*Δ*^*/Y* testes samples collected at P20 are consistent with this model, and proteins involved in integrin-mediated cell adhesion^44^, cytoskeleton remodeling^45^,^46^, cystic fibrosis transmembrane conductance regulator folding and maturation^47^, water homeostasis transport^48^, tumor necrosis factor-α-induced apoptosis, and survival^49^,^50^ that were identified exhibited the most dynamic expression profiles. Defects in any of these pathways would cause male infertility, thereby supporting the hypothesis that CUL4B is critical for coordinating the structural factors that are required for subcellular organelle transformation and survival in the initial post-meiotic spermatids. In particular, significant differences were observed in the expression of pathways involved in cellular morphogenesis related to development of the acrosome structure and condensation of the nucleus. Furthermore, a process networks analysis confirmed that proteins involved in rearrangements of the cytoskeleton and actin filaments^46^, proteins mediating folding in the nucleus, endoplasmic reticulum, and cytoplasm, and proteins mediating ubiquitin-proteasomal proteolysis^9^ were notably dynamic as well, and these proteins have significant roles in spermiogenesis by facilitating the morphological changes that round spermatids undergo. Based on the expression pattern obtained for CUL4B during post-meiotic germ cell development, expression of CUL4B was first detected in stage 4 round spermatids near the end of the Golgi phase and at the onset of the acrosome phase. At the start of spermiogenesis, round spermatids undergo reconstruction of their chromatin and condensation of DNA. During this stage, the canonical histones, H2A, H2B, H3, and H4, help modify chromosome structure and the packaging of DNA into nucleosomes. Histone H3 is the most important histone for the latter and it is also involved in epigenetic regulation during the S phase of the cell cycle. In mammals, histone H3 has three isoforms: the canonical histones, H3.1 and H3.2.3, and the non-canonical histone, H3.3. The latter is encoded by two genes (*H3f3a* and *H3f3b*) that encode two identical proteins, H3.3A and H3.3B, yet these differ in their nucleotide sequences^51^. Unlike the canonical histones, H3.3 is also replication-independent and is expressed throughout the cell cycle. H3.3 is deposited at promoter regions, transcribed genes, regulatory elements, and enhancers to provide a more accessible nucleosome structure^52^. Furthermore, H3.3 is expressed in mitotic, meiotic, and post-meiotic male germ cells and plays an important role in the regulation of genome function and stability^53^. In *Drosophila*, H3.3-deficiency leads to male sterility and chromatin defects in male germ cells^43^. In mice, H3.3a deficiency leads to male mice that are subfertile with abnormal spermatozoa, while the infertility of H3.3b heterozygous male mice involves an arrest in the generation of round spermatids and the production of sperm with head defects^53^. In previous studies, CUL4B was found to participate in various posttranslational modifications of histones via monoubiquitination or polyubiquitination, and also contributes to the response to radiation-induced DNA damage, with histones H2A, H3, and H4 becoming substrates of CUL4B^54^. In the immunoblotting data of the present study, lower levels of H3.3 expression were detected in P20 testes tissues of *Cul4b*^*∆*^*/Y* male mice compared to WT male mice. Immunofluorescence staining of *Cul4b*^*∆*^*/Y* sperm also showed abnormal heads with disintegrated nuclei or a loss of nuclei. During the round spermatid stage, upregulation of transcriptional activity is a requirement for spermiogenesis-related processes^55^. Thus, loss of H3.3 could potentially lead to reduced transcriptional activation and down-regulation of H3.3-related genes, thereby compromising the stages of spermiogenesis^53^. Taken together, these data support a role for CUL4B in mediating a reduction of stage 4 round spermatids and a deficiency in acrosome formation and the onset of nuclear morphogenesis.

A pharmacologic approach to hormonal and non-hormonal male contraception remains a long-term challenge in medicine^56^. Non-hormonal male contraceptive approaches have the advantage that hormones or hormone blockers are not administered, and the safety and effectiveness of these approaches need to be further tested^57^. Ideally, useful contraceptive compounds should provide a complete and reversible treatment of infertility in the testes, and this may include addressing defective acrosome formation, irregular chromatin remodeling, misregulated cytoskeletal changes, and premature spermiation^58^. In the present study, disruption of *Cul4b* in a mouse model mainly affected the round spermatids and resulted in reduced sperm counts and motility without affecting hormone levels, mitosis, and meiosis. Therefore, it is proposed that CUL4B represents a promising target for the development of non-hormonal male contraceptives. The results of the proteomic analysis performed in the present study may further facilitate the identification and characterization of additional testes protein targets for non-hormonal male contraceptives, and/or for the treatment of male fertility.

Proteome profiles have been established for growing gonocytes, differentiating spermatogonia, meiotic spermatocytes, and for post-meiotic spermatid development involved in normal mouse spermatogenesis^7^,^59^. To our knowledge, the systematic quantitative proteomics analysis of WT and *Cul4b*^*Δ*^*/Y* testes tissues at P20 in the present study is the first to demonstrate that disrupted expression of the E3 ligase, CUL4B, in testes tissues affects the expression of the Cullin-RING ubiquitin ligase network, and it creates a reference dataset for the study of global interactions between proteins involved in spermatogenesis^37^,^60^.

In conclusion, we successfully used CUL4B-deficient mice to investigate the molecular mechanisms of fertility, and the insights obtained may also be applicable to humans. In particular, a critical role for X-linked CUL4B in regulating both canonical and non-canonical histones that affect the round spermatid stage was demonstrated. In addition, CUL4B was found to participate in acrosomal formation and the regulation of nuclear condensation during spermiogenesis. These findings enhance our understanding of the molecular basis of CUL4B in spermiogenesis, and they have the potential to facilitate the development of therapeutic strategies for male infertility, as well as the development of non-hormonal male contraceptives.

## Materials and Methods

### Animals

*Cul4b*^*lox*^*/Y* male mice and *Cul4b*^*lox/+*^ female mice carrying a *loxP*-floxed *Cul4b* allele were generated as described previously^24^. All of the mice used in this study were serially backcrossed to a C57BL/6 (B6) genetic background for at least ten generations. To acquire surviving *Cul4b* mutant mice, *Cul4b* was selectively inactivated in embryonic tissues. Floxed (*Cul4b*^*lox/+*^ or *Cul4b*^*lox/lox*^) female mice were mated with *Sox2-Cre* transgenic male mice (Jackson Laboratory, Bar Harbor, ME, USA) to produce CRE activity in the epiblast at embryonic day 6.5^61^. Mice that inherited both the *Cre* transgene and floxed *Cul4b* allele were either hemizygous (*Cul4b*^*Δ*^*/Y* or *Cul4b*^*lox*^*/Y; Sox2-Cre*) or heterozygous (*Cul4b*^*Δ/+*^ or *Cul4b*^*lox/+*^; *Sox2-Cre*) for the *Cul4b*-deleted allele. After genotyping for the general or modified *Cul4b* and *Cre, Cul4b*^*+*^*/Y* and *Cul4b*^*lox*^*/Y* littermates were used as the wild-type and control groups. Food and water was provided *ad libitum*, and standard care was provided according to laboratory animal care policies. All experimental procedures were approved by Institutional Animal Care and Use Committee (IACUC) of National Taiwan University. All experimental methods were performed in accordance with the approved guidelines. All efforts were made to minimize animal suffering.

### Mating phenotypes

To assess male sterile phenotypes, controlled breeding experiments were conducted and the number of pups sired were recorded. Sexually mature *Cul4b*^*+*^*/Y, Cul4b*^*lox*^*/Y*, and *Cul4b*^*Δ*^*/Y* mice (P80) were mated with mature wild-type females (*Cul4b*^*+/+*^, B6 strain). Each male mouse was paired with at least two females for a period of two months, or until a litter was born. Within these two months, each female that had a copulatory plug would be isolated and checked for visible signs of pregnancy. If a female had not mated with the male within four days during their estrus cycle, the female was replaced with another female. The total number of litters and pups were recorded for each male, and the average litter sizes were subjected to statistical analysis.

### Hormone assays

Hormone concentrations were measured in individual serum samples collected from mice at P80. Briefly, whole blood was collected in non-heparinized tubes from the retro-orbital venous plexus and was allowed to clot for 30 min at room temperature (RT). Serum samples were isolated by centrifugation at 1,000× *g* for 10 min at 4 °C and then were stored at −80 °C. Testosterone levels and follicle-stimulating hormone (FSH) levels were quantitatively determined with ELISA kits (DB52181; IBL, Hamburg, Germany and ERK-R7007; Endocrine Technologies, Newark, CA, USA). A standard curve was obtained from standard sample concentrations that were provided by the manufacturer.

### Sperm count and motility

To evaluate epididymal sperm count and motility, fresh cauda epididymides were dissected from P80 male mice and were placed into 2 ml of M16 medium (Sigma-Aldrich, St. Louis, MO, USA) supplemented with 1% bovine serum albumin (BSA; Sigma-Aldrich) and maintained at 37 °C and 5% CO_2_. Each epididymis was minced, thereby allowing the spermatozoa to disperse into the medium. After 30 min, the sperm suspension was collected and diluted for quantitative assessment. Concentration (10^6^/ml) and motility (%) were measured using a computer-assisted sperm analysis (CASA) device with integrated visual optical system (IVOS) software (Hamilton-Thorne Research, Beverly, MA, USA). Videos of motile spermatozoa were captured using an Olympus IX-71 microscope fitted with an Olympus DP71 cooled charge-coupled device (CCD) digital camera (Olympus Optical Co. Ltd, Tokyo, Japan).

### Analysis of spermatozoa

To evaluate spermatozoa deformities, epididymal sperm released in M16 medium were centrifuged at 300× *g* for 5 min, then were resuspended in PBS and spread on silane-coated glass slides (Muto Pure Chemicals, Tokyo, Japan). Sperm smears were air-dried, fixed in 4% paraformaldehyde, and then stained with hematoxylin and eosin (H&E; Muto Pure Chemicals) for morphological analysis or were subjected to immunofluorescence. The H&E-stained slides were observed systematically and were classified according to morphological integrity^62^. One hundred spermatozoa from each mouse were evaluated as normal or abnormal under a light microscope at 400× magnification. Defective spermatozoa were further classified as: hookless, having an amorphous head, having a bent neck, being U-shaped, or having a coiled tail. Spermatozoa abnormalities were recorded as percentages of the total number of sperm counted. To evaluate substructure, acrosomes were stained with Alexa Fluor 568-conjugated peanut agglutinin (PNA) lectin (25 μg/ml; Molecular Probes, Invitrogen, Eugene, OR, USA). After 1 h, mid-piece components were labeled with a fluorescent mitochondrial marker, MitoTracker^®^ Green FM (500 nM; Molecular Probes) for 1 h. After washing, the slides were treated with 4′,6-diamidino-2-phenylindole (DAPI; 0.5 μg/ml; Sigma-Aldrich) for 10 min. The final slides were mounted with fluorescent mounting medium (Dako North America, Carpinteria, CA, USA) and were imaged with sequential scanning under each appropriate wavelength with a Leica DMR microscope (Leica Wetzlar, Heidelberg, Germany) equipped with fluorescence illumination and an Olympus DP72 CCD digital camera (Olympus Optical Co. Ltd).

### IVF

Female mice (B6 strain, 5 weeks of age) were injected with pregnant mare serum gonadotropin (PMSG) and human chorionic gonadotropin (hCG) (Sigma-Aldrich), then oocytes were collected and treated with hyaluronidase (1 mg/ml; Sigma-Aldrich) for 5 min to remove cumulus cells. The oocytes were subsequently placed in HTF medium (Quinn’s Advantage Fertilization Medium; SAGE IVF Inc., Trumbull, CT, USA) covered with mineral oil (Sigma-Aldrich). To minimize differences in quality, oocytes from one oviduct were separated into two pools and each pool was fertilized with sperm from *Cul4b*^*+*^*/Y* or *Cul4b*^*Δ*^*/Y* mice.

Epididymal sperm were collected from P80 mice and were added to the oocytes to achieve a final concentration of 1 × 10^6^ sperm/ml. After 6 h at 37 °C under 5% CO_2_, the oocytes were washed and cultured in HTF medium overnight. Fertilized eggs were subsequently counted and scored at the two-cell embryonic stage.

### Histology

Male mice at P1, P5, P15, P20, P27, P35, P42, and P80 were anesthetized and perfused intracardially (3 ml/min) with 0.9% normal saline for 10 min, then with buffered neutral formalin (Sigma-Aldrich) for 15 min. After dissection of the testes and removal of the epididymides, the tissues were post-fixed in modified Davidson’s fixative^63^ for 24 h at 4 °C. The specimens were rinsed with PBS, dehydrated in ascending concentrations of ethanol, and embedded in paraffin wax. Sections (5 μm) were stained with H&E and were mounted on glass slides. The testicular sections were examined systematically to detail the different germ cell types during different stages^2^. The caput, corpus, and cauda portions of the epididymal cross-sections were observed to determine the amount of spermatozoa within the lumen.

### Immunohistochemical staining

Paraffin-embedded sections were deparaffinized in xylene and rehydrated in descending concentrations of ethanol. After being washed in PBS, antigen retrieval was performed in boiling 10 mM sodium citrate solution (pH 6.0) containing 0.05% Tween-20 for 20 min. Sections were subsequently incubated in 3% H_2_O_2_ for 10 min and then in Rodent Block M (Biocare Medical, Concord, CA, USA) at RT. After 1 h, the sections were incubated with primary antibodies diluted in Tris-HCl buffer containing 0.1% Tween and 0.015 M NaN_3_ (Dako Antibody Diluent; Dako North America) for 16 h at 4 °C. Rabbit, mouse, and rat IgG were used as negative controls. Following three washes in PBS containing 0.5% Tween-20 (PBST) for 10 min, the appropriate horseradish peroxidase (HRP)-labeled secondary antibodies were applied for 1 h at RT. To detect mouse or rabbit primary antibodies versus rat or goat primary antibodies, the Super Sensitive Polymer-HRP IHC Detection System (BioGenex, San Ramon, CA, USA) or Histofine Simple Stain MAX PO Kits (Nichirei, Tokyo, Japan) were used, respectively, with 3,3′-diaminobenzidine (DAB) as the chromogen (BioGenex). Nuclei were counterstained with hematoxylin. The sections were dehydrated and mounted with coverslips (Malinol NX; Muto Pure Chemicals).

### Immunofluorescent staining

Paraffin-embedded sections were prepared as described in the previous section. Appropriate secondary antibodies conjugated to Cy3 or Alexa Fluor 488 (1:100; Jackson ImmunoResearch Laboratories, West Grove, PA, USA) were diluted in 3% BSA and were applied in the dark for 1 h at RT. After washing, nuclei were stained with DAPI for 10 min and then the slides were mounted with coverslips and fluorescent mounting medium (Dako North America). Images were captured using a Zeiss LSM 780 inverted confocal laser scanning microscope (Axio Observer Z1; Carl Zeiss, Göttingen, Germany) equipped with a high sensitivity GaAsP and were analyzed using ZEN 2010 software (version 6.0; Carl Zeiss). The antibodies used in this study are listed in Supplementary Table S1.

### TUNEL assay

DNA fragmentation in apoptotic cells in paraffin-embedded sections was detected with the ApopTag Fluorescein *In Situ* Apoptosis Detection Kit (S7110; Millipore, Billerica, MA, USA). Briefly, the sections were deparaffinized, rehydrated, and permeabilized with proteinase K solution (Invitrogen). Then, each section was incubated with the TUNEL mixture for 1 h at 37 °C in the dark. After washing, sections were counterstained with DAPI for 10 min. Positive controls were treated with 3 U/ml DNase I (Promega, Madison, WI, USA) for 20 min before TUNEL labeling was performed, while negative controls were incubated without the TdT enzyme. Fluorescent images were acquired with the same exposure time and the numbers of apoptotic tubules and cells were counted.

### Transmission electron microscopy (TEM)

Testes were pre-fixed in a solution of 4% paraformaldehyde and 2.5% glutaraldehyde for 16 h at 4 °C. Testes were then cut into small pieces and post-fixed in 1% osmium tetroxide for 1 h at RT. After fixation, specimens were dehydrated in a series of ascending concentrations of ethanol solutions and were embedded in epoxy resin (Glycidether 100; Merck, Darmstadt, Germany). Ultrathin sections (~60 nm) were cut with a diamond knife on an LKB Ultrotome (LKB-Produkter AB, Stockholm, Sweden) and were mounted on nickel grids to be stained with 5% uranyl acetate and 5% lead citrate. Sections were examined with a JEM-1400 transmission electron microscope system (JEOL, Tokyo, Japan) at 80 kV that was equipped with an Orius CCD digital camera.

### Proteomic analysis

#### Preparation of samples

Testes were collected, homogenized, and lysed in protein denaturation buffer (7 M urea, 2 M thiourea, 65 mM dithiothreitol) containing 1% v/v protease inhibitor cocktail (Sigma-Aldrich) on ice for 30 min. After centrifugation at 40,000 ×  *g* for 1 h at 4 °C, the total protein concentration of each sample was measured by the Bradford method (Bio-Rad, Hercules, CA, USA) with serially diluted BSA as standards.

#### In solution digestion and peptide purification

The testes lysates were subsequently incubated in a reduction buffer [10 mM DTT in 25 mM ammonium bicarbonate (ABC) for 1 h at 37 °C, and then in an alkylation buffer [55 mM iodoacetamide in 25 mM ABC] for 1 h at RT in the dark. To avoid over-alkylation, excess reducing agent was added to neutralize the unreacted iodoacetamide. The urea concentration was reduced to less 1 M with the addition of 25 mM ABC and the protein samples were digested with trypsin (protein:trypsin = 30:1, g/g; Promega) at 37 °C. After 16 h, the digestive reaction was quenched by acidifying the sample with 0.1% (v/v) formic acid (FA). Digested peptide samples were dried in a vacuum centrifuge and were reconstituted in 0.1% FA. Prior to mass spectrometric analysis, the digested peptides were desalted and concentrated using ZipTip^®^ pipette tips with 0.6 μL C_18_ resin (Millipore). The tips were equilibrated sequentially in pure acetonitrile (ACN), then in 50% ACN (v/v) in 0.1% FA, and then in 0.1% FA before loading the peptides in the tips. The concentrated peptides were subsequently washed with 0.1% FA and eluted in a 50% ACN/0.1% FA solution. After drying the purified peptides under vacuum, the peptides were reconstituted in 0.1% FA and were analyzed by LC-MS/MS.

#### Mass spectrometry analysis

Digested peptide mixtures of total testicular proteins were subjected to NanoLC-nanoESI-MS/MS analysis performed at the Core Facilities for Proteomics Research (Institute of Biological Chemistry, Academia Sinica, Taipei, Taiwan). The mass spectrometry analysis was processed on a nanoAcquity system (Waters, Milford, MA, USA) coupled to a LTQ-Orbitrap Elite hybrid ion trap-orbitrap mass spectrometer (Thermo Fisher Scientific) equipped with a PicoView nanospray interface (New Objective, Woburn, MA, USA). The resulting peptide mixtures were loaded onto a nanoAcquity UPLC BEH130 column (75 μm internal diameter, 25 cm length; Waters) packed with a C_18_ resin containing 1.7 μm particles (pore size, 130 Å) in 0.1% FA in water (solvent A). Peptides were separated using a segmented gradient of 5–40% solvent B (ACN with 0.1% FA) at 300 nl/min. The mass spectrometer was operated in a data-dependent acquisition mode. Full survey mass spectra scans of mass/charge ratio (m/z) 350–1600 were acquired with a resolving power of 120,000 at 400 m/z and an automatic gain control (AGC) target of 10^6^. The twenty most intense ions were sequentially selected for collision-induced dissociation MS/MS fragmentation in a linear ion trap (AGC target value: 10,000) with previously preferred ions dynamically excluded for 60 s. Ions with single and unrecognized charge states were also excluded.

#### Label-free peptide quantification and identification

Quantitative analysis of the MS/MS data was performed with Progenesis QI for Proteomics (QIP) software (Nonlinear Dynamics, Newcastle, UK). To compensate for drifts in retention time, one sample was set as an alignment reference and all other samples within this trial were automatically aligned. Matching features with one charge and two or fewer isotopes were deleted from further analyses. Normalization factors for each sample were used to correct experimental variations. Statistical analysis was performed using a between-subject design. *P*-values were calculated by a repeated measures analysis of variance (ANOVA) test. MS/MS spectra data files from LTQ-Orbitrap were subsequently converted to Mascot Generic format (MGF files) to perform a search of the Mascot Daemon server (version 2.2; Matrix Science, London, UK) against a target protein sequence database with the following parameters: peptide mass tolerance, 10 ppm; fragment mass tolerance, 0.6 Da; two trypsin missed cleavages allowed; and variable modifications of carbamidomethylation and oxidation allowed. A Mascot integrated decoy database search was used to adjust the false discovery rate to 1%, and searching was performed with an ion score cut-off (Mascot score) of 26. The significance threshold was set at *P* < 0.05. Non-redundant protein sets were determined from the output XML files. Differentially expressed proteins between the WT and mutant mice testes samples from P20 are listed in Supplementary File S1.

### Bioinformatics analysis

Identified proteins were subjected to a hierarchical clustering analysis and are presented as a heat map. Each identified protein value at the same age including two groups was loaded into the Cluster 3.0 software and the protein spots were subjected to k-means clustering, hierarchical clustering, and self-organizing maps. The clustering results were viewed using TreeView software to show proteomic profiles of differentially expressed proteins. Pathway analyses using verified literature databases were performed with MetaCore software (GeneGO Inc., St. Joseph, MI, USA).

### Immunoblotting

Testes were collected at P20 mice. Briefly, testes were homogenized with tissue protein extraction reagent (T-per, Thermo Scientific, Waltham, MA, USA) containing 1% protease inhibitor cocktail (P8340, Sigma-Aldrich), according to the manufacturer’s instructions. Protein concentrations were determined using a Pierce BCA protein assay kit (Thermo Fisher Scientific). Protein samples were separated by SDS-PAGE and electrotransferred to polyvinylidene fluoride membranes. Bound proteins were detected using the indicated primary antibodies (see Supplementary Table S1 online), and were visualized using HRP-conjugated secondary antibodies and Immobilon Western Chemiluminescent HRP substrate (WBKLSO500, Millipore). Bands were semiquantified with Multi Gauge V3.0 software (Fujifilm, Tokyo, Japan) and were normalized against α-tubulin.

### Statistical analysis

All data were analyzed with GraphPad Prism 5 (GraphPad Software, San Diego, CA, USA) and are presented as the mean ± SEM for each genotype. A two-tailed unpaired Student’s *t*-test or one-way analysis of variance (ANOVA) followed by *post hoc* Bonferroni’s multiple comparison tests were performed to compare data. A *P*-value less than 0.05 was considered statistically significant.

## Additional Information

**How to cite this article**: Lin, C.-Y. *et al*. Human X-linked Intellectual Disability Factor CUL4B Is Required for Post-meiotic Sperm Development and Male Fertility. *Sci. Rep.* **6**, 20227; doi: 10.1038/srep20227 (2016).

## Supplementary Material

Supplementary Information

Supplementary Video S1

Supplementary File S1

## Figures and Tables

**Table 1:** Quantification of the tubules entering meiosis and post-meiosis in the first wave of spermatogenesis.

**Postnatal Day**	***Cul4b***^***+***^***/Y*****% (n)**	***Cul4b***^***Δ***^***/Y*****% (n)**
Meiotic entry rate^a^		
P1	0 (396)	0 (323)
P5	0 (344)	0 (408)
P15	80 (1307)	83 (1334)
P20	96 (837)	99 (870)
P27	98 (585)	98 (714)
P35	100 (372)	98 (439)
P42	100 (388)	100 (404)
Post-meiotic entry rate^b^		
P1	0 (174)	0 (184)
P5	0 (192)	0 (192)
P15	0 (229)	0 (213)
P20	94 (797)	91 (738)
P27	98 (706)	97 (752)
P35	94 (460)	93 (457)
P42	96 (376)	96 (442)

Five mice of each genotype were analyzed at each of the indicated timepoints.

^a^Number of SCP3-positive tubules divided by the total number of tubules.

^b^Number of PNA lectin-positive tubules divided by the total number of tubules.
