# Supplementary material for: Human X-linked Intellectual Disability Factor CUL4B Is Required for Post-meiotic Sperm Development and Male Fertility
Source: Sci Rep. 2016 Feb 2;6:20227. doi: 10.1038/srep20227 (PMC4735749; doi:10.1038/srep20227)
Supplement: Supplementary Information [file srep20227-s1.pdf]

Supplementary Information

Title

Human X-linked Intellectual Disability Factor CUL4B Is Required for Post-meiotic Sperm Development and Male Fertility

Authors

Chien-Yu Lin[1*], Chun-Yu Chen[1*], Chih-Hsiang Yu[1], I-Shing Yu[2], Shu-Rung Lin[3], June-Tai Wu[4] Ying-Hung Lin[5], Pao-Lin, Kuo[6,7], Jui-Ching Wu[1#], Shu-Wha Lin[1,8,9#]

# These authors contributed equally to this work

* These authors also contributed equally to this work

Figure S1. Reproductive organs and hormone levels in adult male mice. Reproductive organs and serum were harvested from adult (P80) male mice to analyze gonadal development. (A) Macroscopic features of the reproductive system. *Cul4b*$^{\Delta}$*/Y* (*Δ/Y*) mice showed no visible morphological differences from WT control mice (*Cul4b*$^{+}$*/Y*; *+/Y* and *Cul4b*^{lox}*/Y*; *lox/Y*). Abbreviations: T, testis; E, epididymis; V, vas deferens; B, bladder; SV/CG, seminal vesicle/coagulating gland. Scale bar, 1 cm. (B) Isolated organs were weighed and normalized against total body weight. No significant differences in the normalized weights of the testes, epididymides, and seminal vesicles were observed between the *Cul4b*$^{\Delta}$*/Y* and WT control mice. (ANOVA test; ns, not significant; n = 11/group). (C,D) Serum levels of testosterone (C) and FSH (D) were measured and there were no significant differences between the three different genotypes. (ANOVA test; ns, not significant; n = 5/group).

Figure S2. Spermatozoa within the epididymal lumen. Representative images from H&E-stained caput, corpus, and cauda portions of epididymal cross-sections obtained from P80 mice. The amount of spermatozoa within the *Cul4b*$^{\Delta}$*/Y* (*Δ/Y*) epididymal lumen was greatly

reduced compared to the *Cul4b*$^{+}$*/Y* (*+/Y*) and *Cul4b*^{lox}*/Y* (*lox/Y*) lumens. Scale bar, 50 µm.

Figure S3. Morphology of spermatozoa from the cauda epididymides. Epididymal spermatozoa were stained with H&E to examine their structural composition. (A) Spermatozoa from WT control mice (*Cul4b*$^{+}$*/Y*; *+/Y* and *Cul4b*^{lox}*/Y*; *lox/Y*) had typical hook-shaped heads and linear morphology. (B) Spermatozoa from *Cul4b*$^{\Delta}$*/Y* mice were structurally defective, particularly in regard to the acrosome and nucleus. Abbreviations: a, acrosome; n, nucleus; c, connecting piece; m, mid-piece; p, principle piece; e, end piece. Scale bar, 2 µm.

Figure S4. *In vitro* fertilization (IVF) with epididymal sperm. Super-ovulated WT oocytes were collected from B6 females; sperms were isolated from the cauda epididymides of *Cul4b*$^{+}$*/Y* (*+/Y*) and *Cul4b*$^{\Delta}$*/Y* (*Δ/Y*) males. (A) On day 1 following IVF, representative images of two-cell stage embryos (arrows) and non-dividing oocytes (arrowheads) were collected. Scale bar, 100 µm. (B) The success rate of IVF is shown in the bar graph. The number of two-cell stage embryos were divided by the total number of oocytes. *Cul4b*$^{\Delta}$*/Y* sperm were associated with fewer fertilization events compared to the *Cul4b*$^{+}$*/Y* sperm. Data are representative of 625 and 547 oocytes that were fertilized with sperm collected from *Cul4b*$^{+}$*/Y* and *Cul4b*$^{\Delta}$*/Y* mice (n = 6 each), respectively. All values are the mean ± SEM. ** $P < 0.01$, Student's *t*-test.

Figure S5. Cross-sections of seminiferous tubules. (A,B) H&E-stained cross-sections of *Cul4b*$^{+}$*/Y* (*+/Y*) and *Cul4b*$^{\Delta}$*/Y* (*Δ/Y*) testes. Roman numerals indicate the stages of the seminiferous tubules. Morphologically normal spermatogenesis was observed in the *Cul4b*$^{+}$*/Y* sections, while the *Cul4b*$^{\Delta}$*/Y* sections exhibited an unusually greater number of empty lumens. Scale bar, 50 µm. (C,D) Quantification of seminiferous tubule count and tubular diameter. There was no significant differences in for either data set between the two genotypes (Student's *t* test; ns, not significant, 6 sections and 100 tubules/mouse, n = 6/group).

Figure S6. Organization of the Sertoli cells and spermatocytes. (A,B) GATA1 and GATA4 immunostaining (brown) revealed the presence of Sertoli cell nuclei at the edge of the tubules in *Cul4b*$^{+}$*/Y* (*+/Y*) mice and *Cul4b*$^{\Delta}$*/Y* (*Δ/Y*) mice, suggesting that Sertoli cell organization is not affected by CUL4B depletion. The number of Sertoli cells within the seminiferous tubules was

also comparable between the two genotypes. (C,D) SCP1 and SCP3 immunostaining (brown) indicated a lack of visible influence of CUL4B deletion on spermatocyte organization. The number of spermatocytes within each set of seminiferous tubules was not significantly affected. Scale bar, 20 µm. All values are the mean ± SEM. (Student's *t*-test; ns, not significant; 30 tubules/mouse; n = 6/group).

Figure S7. Apoptosis in adult mice testicular sections. (A,B) Fluorescent images of TUNEL-positive tubules and cells. Scale bar, 100 µm. (C) The proportion of TUNEL-positive tubules was significantly greater in the $Cul4b^{\Delta}/Y$ mice than in the $Cul4b^{+}/Y$ mice. (D) Quantification of the TUNEL-positive cells observed within 3 visual fields at 100× magnification. A greater number of apoptotic cells were counted in the $Cul4b^{\Delta}/Y$ sections than in the $Cul4b^{+}/Y$ sections. All values are presented as the mean ± SEM. (Student's *t*-test; $^{***}P <$ 0.001; n = 6/group).

Figure S8. TUNEL analysis during the first wave of spermatogenesis. Fluorescent images of TUNEL-positive tubules and cells in testicular sections obtained from P1 to P42. There was not a significant number of germ cells undergoing apoptosis in the testis of the $Cul4b^{\Delta}/Y$ (*Δ/Y*) mice compared to the age-matched $Cul4b^{+}/Y$ (*+/Y*) mice from P1-P20. However, a greater number of apoptotic events were detected in the testis of the $Cul4b^{\Delta}/Y$ mice compared to the $Cul4b^{+}/Y$ mice from P27-P42. Scale bars, 50 µm (P1-P20); 100 µm (P27-P42).

Figure S9. Histology of testes sections prepared from P27 mice. (A) Seminiferous tubules are shown with acrosome signals (red) and TUNEL-positive apoptotic cells (green). DAPI-stained nuclei (blue) were present at the border of the tubules. A visible increase in the number of apoptotic tubules was observed in the $Cul4b^{\Delta}/Y$ (*Δ/Y*) cross-sections. Tubules with steps 1-4 round spermatids (arrows) and steps 5-8 round spermatids (arrowheads) are shown. Scale bar, 50 µm. (B) A quantitative analysis of TUNEL-positive tubules in P27 testes. The distribution of seminiferous tubules (white bars) is presented as the percentage of the total number of tubules. There was no significant difference in the proportion of tubules according to the specific step spermatids between the two genotypes. In contrast, the percentage of TUNEL-positive tubules (gray bars) associated with the steps 5-8 round spermatids was significantly increased in the

Cul4b$^{\Delta}$*/Y* mice compared to the *Cul4b*$^{+}$*/Y* (*+/Y*) mice. All values are presented as the mean ± SEM. (Student's *t*-test; $^{***}P < 0.001$; n = 6/group).

Figure S10. Immunoblotting to detect protein levels of canonical and variant histones in wild-type and CUL4B-deficient testes at P15. Similar protein levels of (A) testis-specific histone H3.3, (B) histones H4 and (C) H3, were detected in *Cul4b*$^{lox/}$*Y* (*lox/Y*) and *Cul4b*$^{\Delta/}$*Y* (*Δ/Y*) testes extracts. Detection of tubulin was used as a loading control. Densitometry values are indicated with the levels of histones H3.3, H4, and H3 and the average signal of the *lox/Y* extract is set to 1. ns, not significant.

Table S1. A summary of primary antibodies used for immunostaining and immunoblotting.

**Antibody**	**Catalog No.**	**Source**	**Dilution**
CUL4B	12916-1-AP	Proteintech	1:100
GATA1	sc-266	Santa Cruz	1:50
GATA4	sc-9053	Santa Cruz	1:100
SCP1	ab15090	abcam	1:200
SCP3	ab97672	abcam	1:200
H3F3B	H00003021-M01	Abnova	1:1000
Histone H3	ab1791	abcam	1:1000
Histone H4	ab10158	abcam	1:1000
α-Tubulin	ab7291	abcam	1:1000
anti-mouse IgG	AP124P	Millipore	1:10000
anti-rabbit IgG	AP132P	Millipore	1:10000

Table S2. Quantification of post-meiotic germ cells from adult mice testicular sections.

**Genotype**	***Cul4b^+/Y***	***Cul4b^Δ/Y***
Round spermatids		
Steps 1-4 (Stages I-IV)	98.80 ± 0.35	97.81 ± 1.06
Steps 5-8 (Stages V-VIII)	100.0 ± 3.33	80.15 ± 2.28**
Elongating spermatids		
Steps 9-10 (Stages IX-X)	97.85 ± 2.62	76.48 ± 5.34*
Steps 11-12 (Stages XI-XII)	99.32 ± 4.68	73.72 ± 2.69**
Elongated spermatids		
Steps 13-14 (Stages I-IV)	95.48 ± 1.72	61.98 ± 2.80***
Steps 15-16 (Stages V-VIII)	99.33 ± 4.67	64.05 ± 3.43**

All values were means ± SEM. Significant differences were performed between the two genotypes (Student's t-test; *, $P<0.05$; **, $P<0.01$; ***, $P<0.001$; 20 tubules of each stage bracket/mouse, n = 6/group).

File S1. Differentially expressed proteins between wild-type and mutant mice testes samples collected at P20 and P27.

Video S1. Motility of WT control and *Cul4b*$^{\Delta}$*/Y* sperm. Sperm were collected from the cauda epididymides, were placed in M16 medium, and were observed with a light microscope. *Cul4b*$^{+}$*/Y* (+/*Y*) and *Cul4b*^{lox}*/Y (lox/Y*) sperm exhibited typical concentrations and motility. In contrast, *Cul4b*$^{\Delta}$*/Y* (*Δ/Y*) sperm were present at a lower concentration and were less motile.

Figure S1

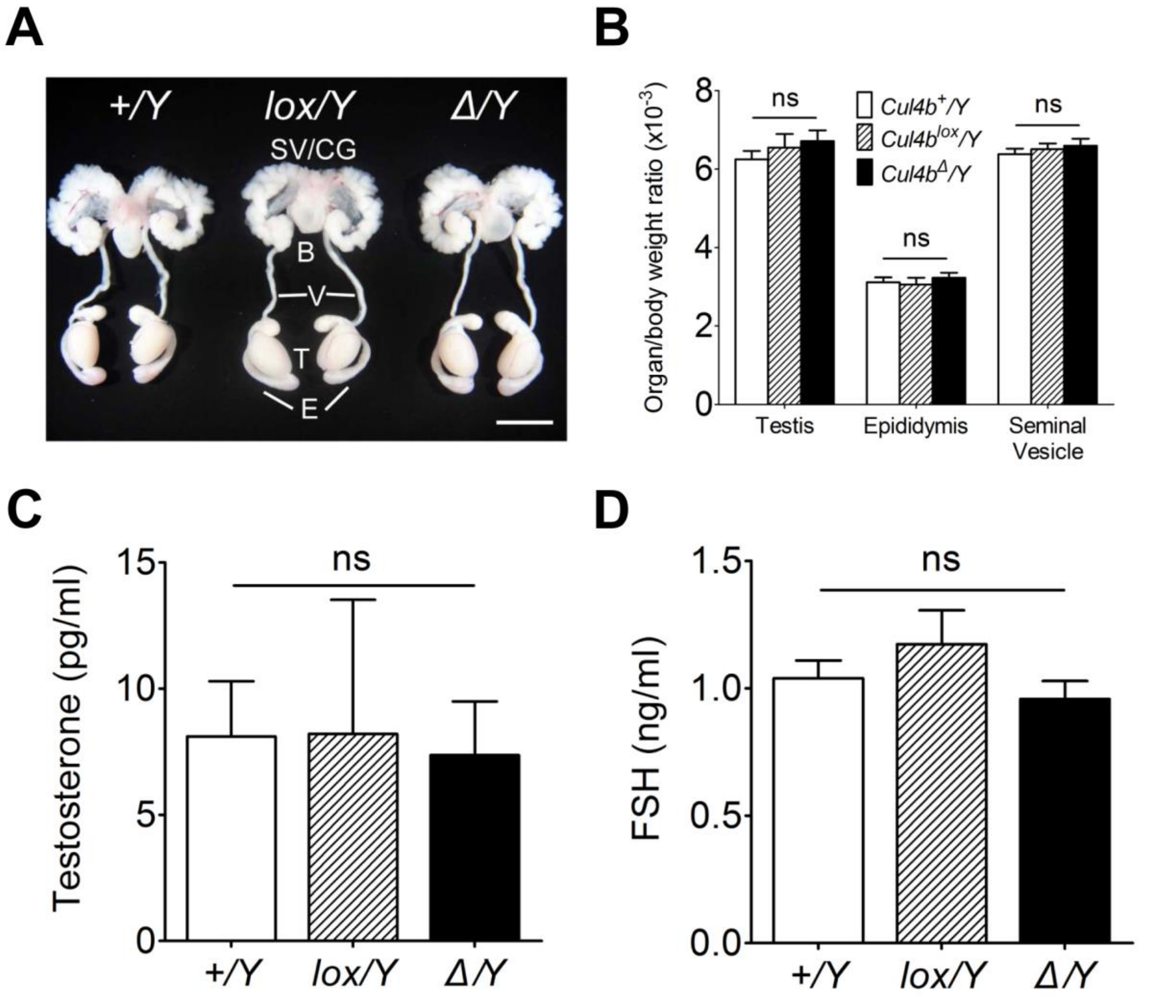
A
+/Y
lox/Y
Δ/Y
SV/CG
B
V
T
E
B
Organ/body weight ratio (x10^{-3})
ns
Cul4b^{+}/Y
Cul4b^{lox}/Y
Cul4b^{Δ}/Y
Testis
Epididymis
Seminal Vesicle
C
Testosterone (pg/ml)
ns
+/Y
lox/Y
Δ/Y
D
FSH (ng/ml)
ns
+/Y
lox/Y
Δ/Y

Figure S2

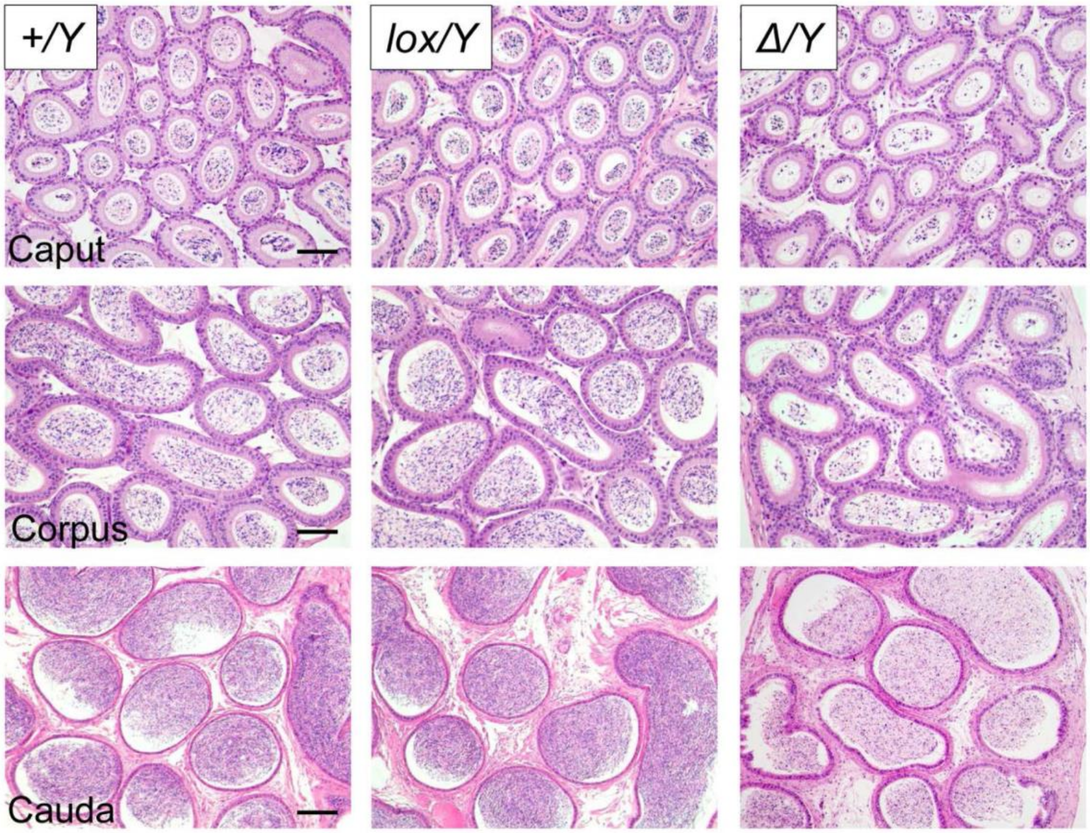

Figure S3

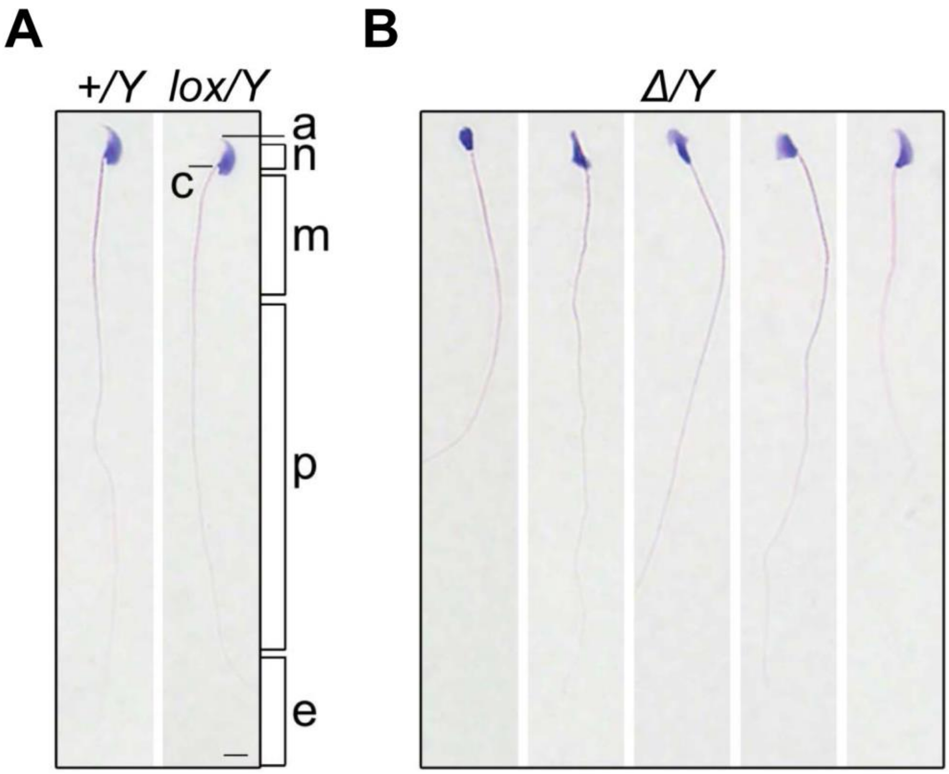

Figure S4

A

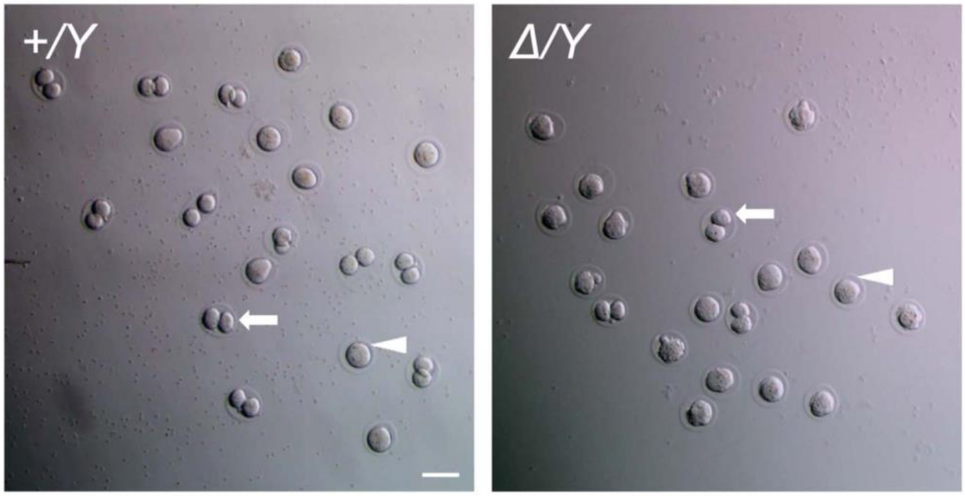

B

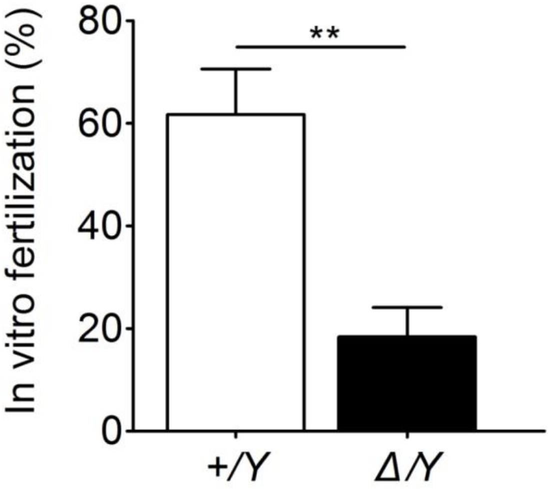

Figure S5

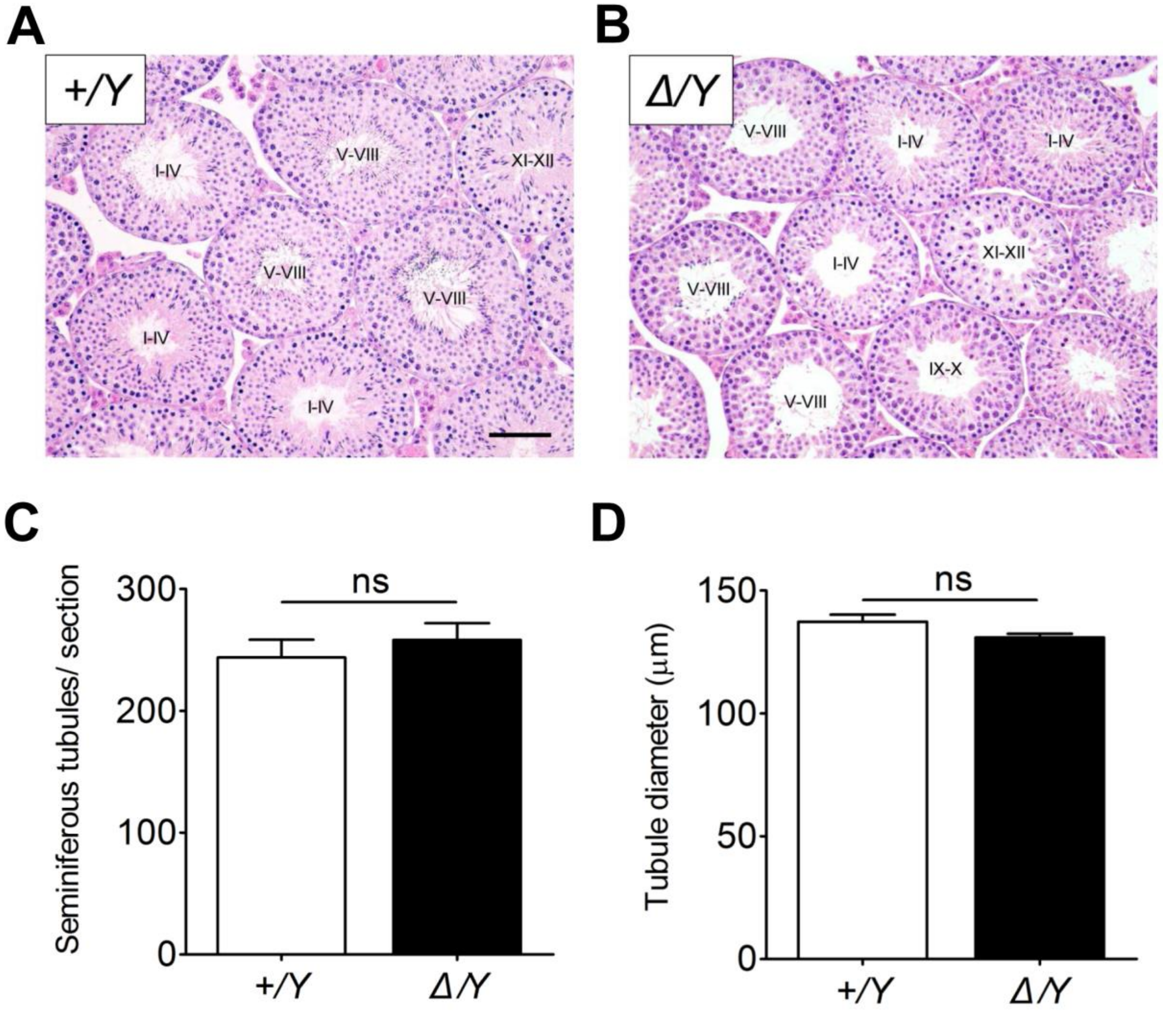
A
+/Y
I-IV
V-VIII
XI-XII
V-VIII
V-VIII
I-IV
I-IV
B
Δ/Y
V-VIII
I-IV
I-IV
I-IV
XI-XII
V-VIII
IX-X
V-VIII
C
Seminiferous tubules/ section
300
200
100
0
ns
+/Y
Δ/Y
D
Tubule diameter (μm)
150
100
50
0
ns
+/Y
Δ/Y

Figure S6

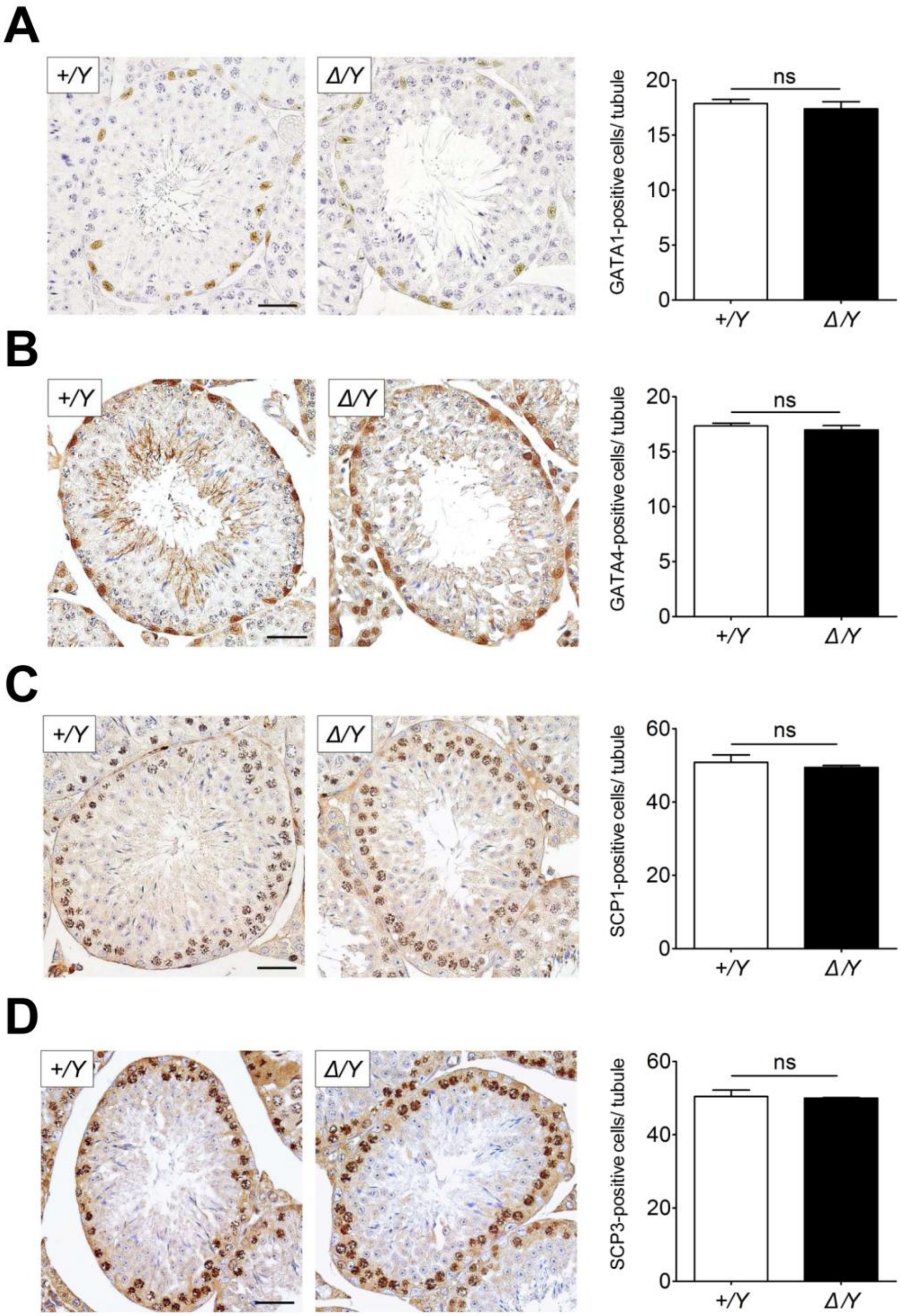
A
+/Y
Δ/Y
ns
GATA1-positive cells/ tubule
20
15
10
5
0
+/Y
Δ/Y
B
+/Y
Δ/Y
ns
GATA4-positive cells/ tubule
20
15
10
5
0
+/Y
Δ/Y
C
+/Y
Δ/Y
ns
SCP1-positive cells/ tubule
60
40
20
0
+/Y
Δ/Y
D
+/Y
Δ/Y
ns
SCP3-positive cells/ tubule
60
40
20
0
+/Y
Δ/Y

Figure S7

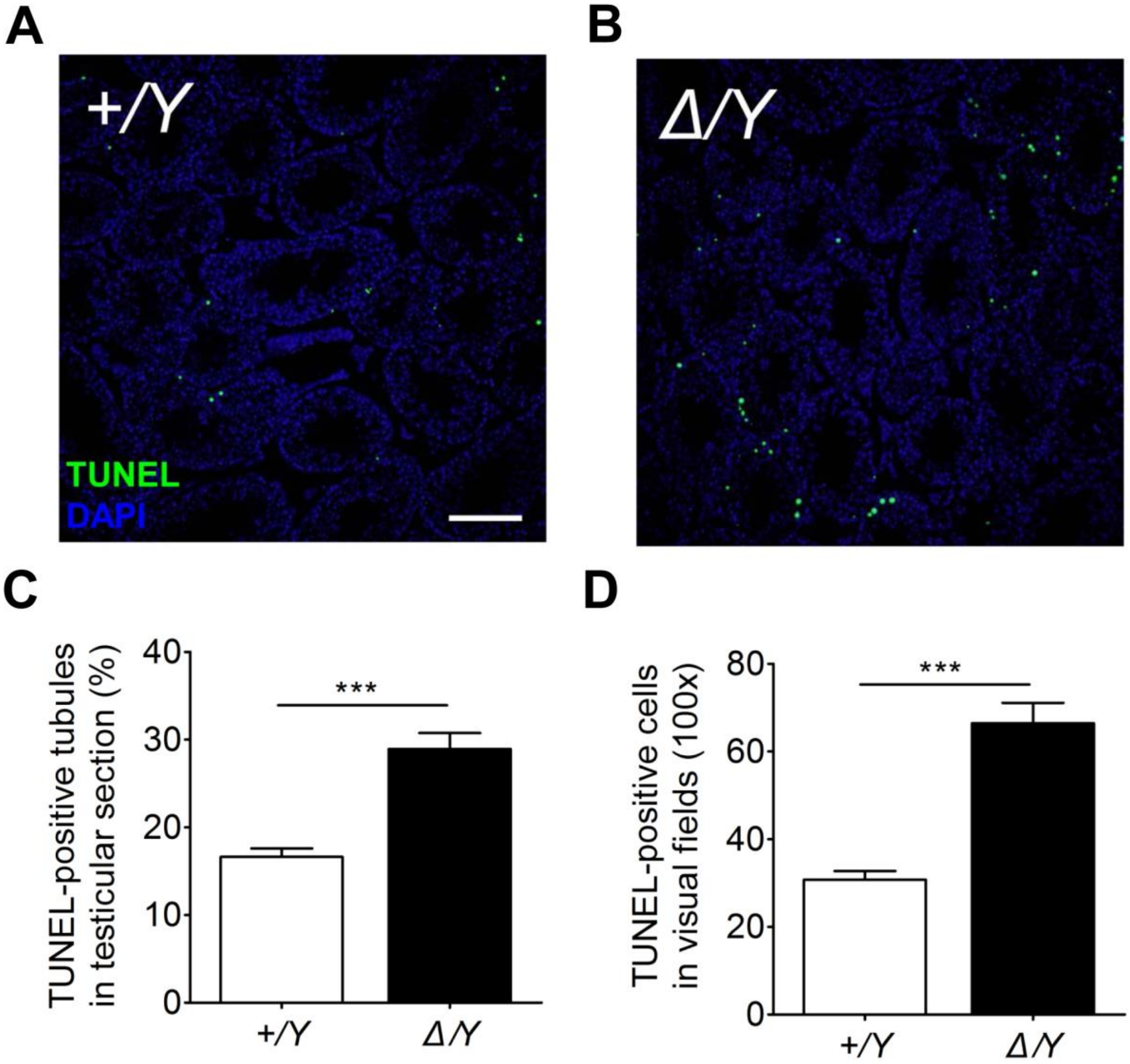
A
B
+/Y
Δ/Y
TUNEL
DAPI
C
D
TUNEL-positive tubules in testicular section (%)
40
30
20
10
0
***
+/Y
Δ/Y
TUNEL-positive cells in visual fields (100x)
80
60
40
20
0
***
+/Y
Δ/Y

Figure S8

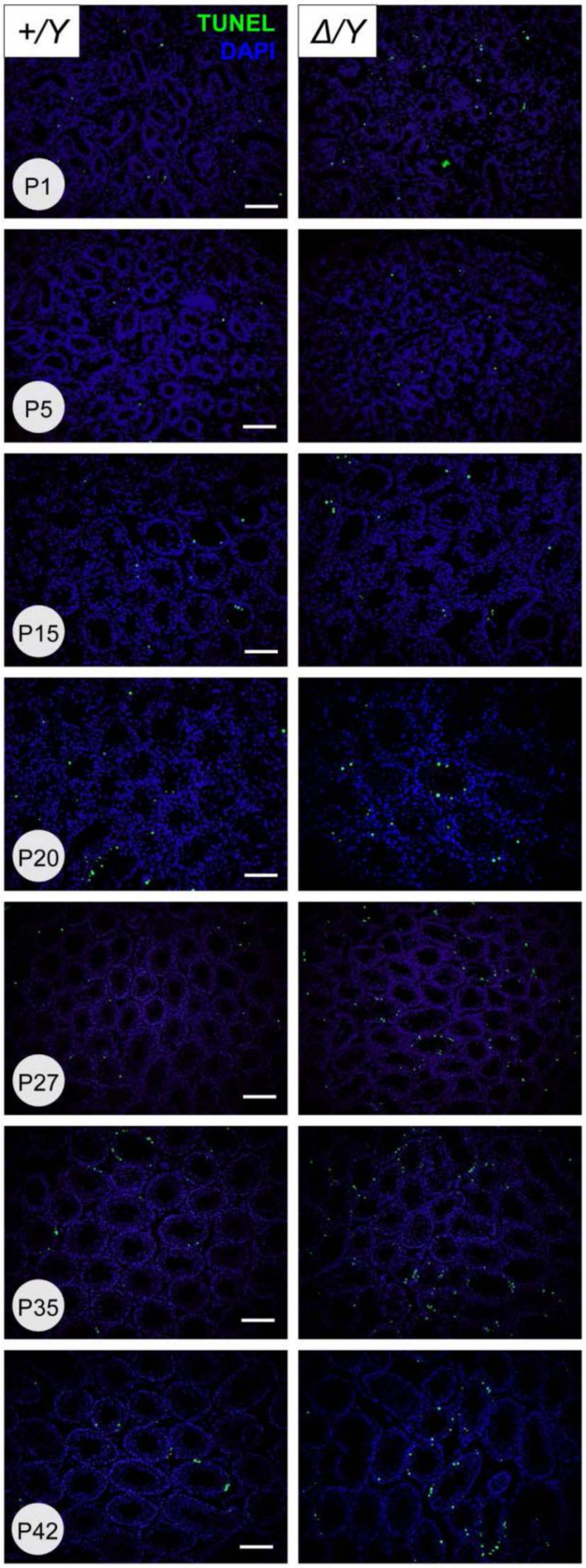

Figure S9

A

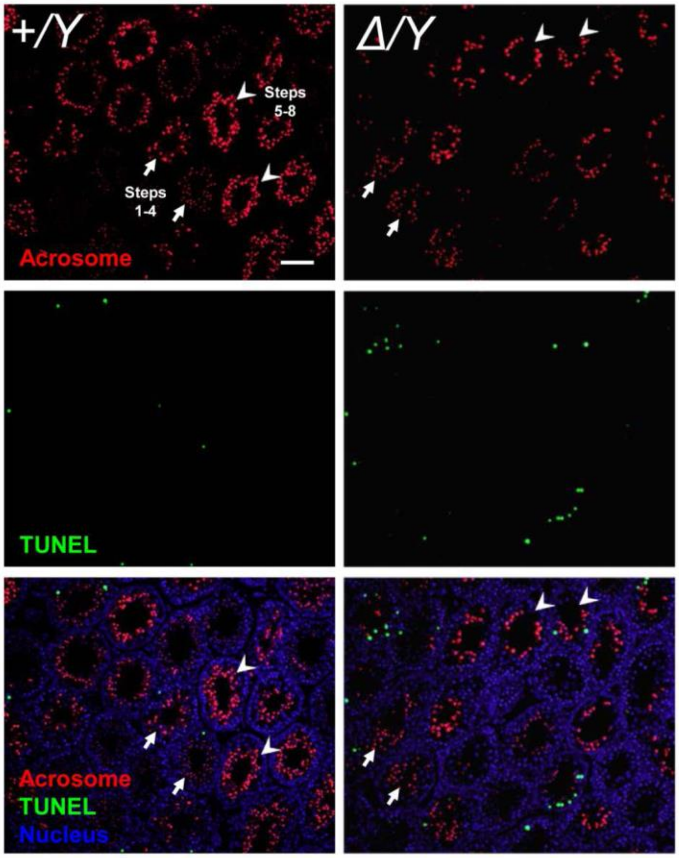
+/Y
Δ/Y
Steps 5-8
Steps 1-4
Acrosome
TUNEL
Acrosome
TUNEL
Nucleus

B

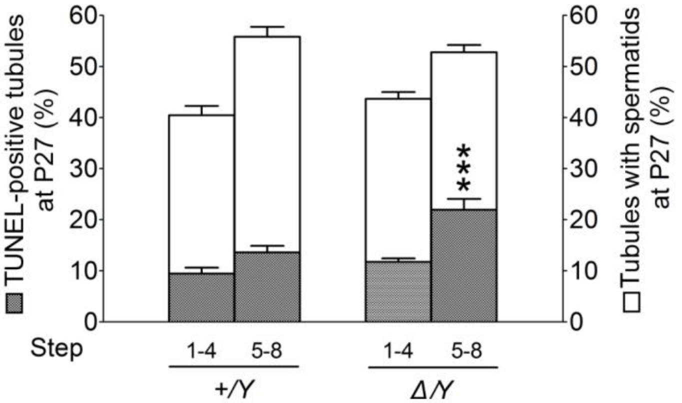
TUNEL-positive tubules at P27 (%)
Tubules with spermatids at P27 (%)
0
10
20
30
40
50
60
***
Step
1-4
5-8
1-4
5-8
+/Y
Δ/Y

Figure S10

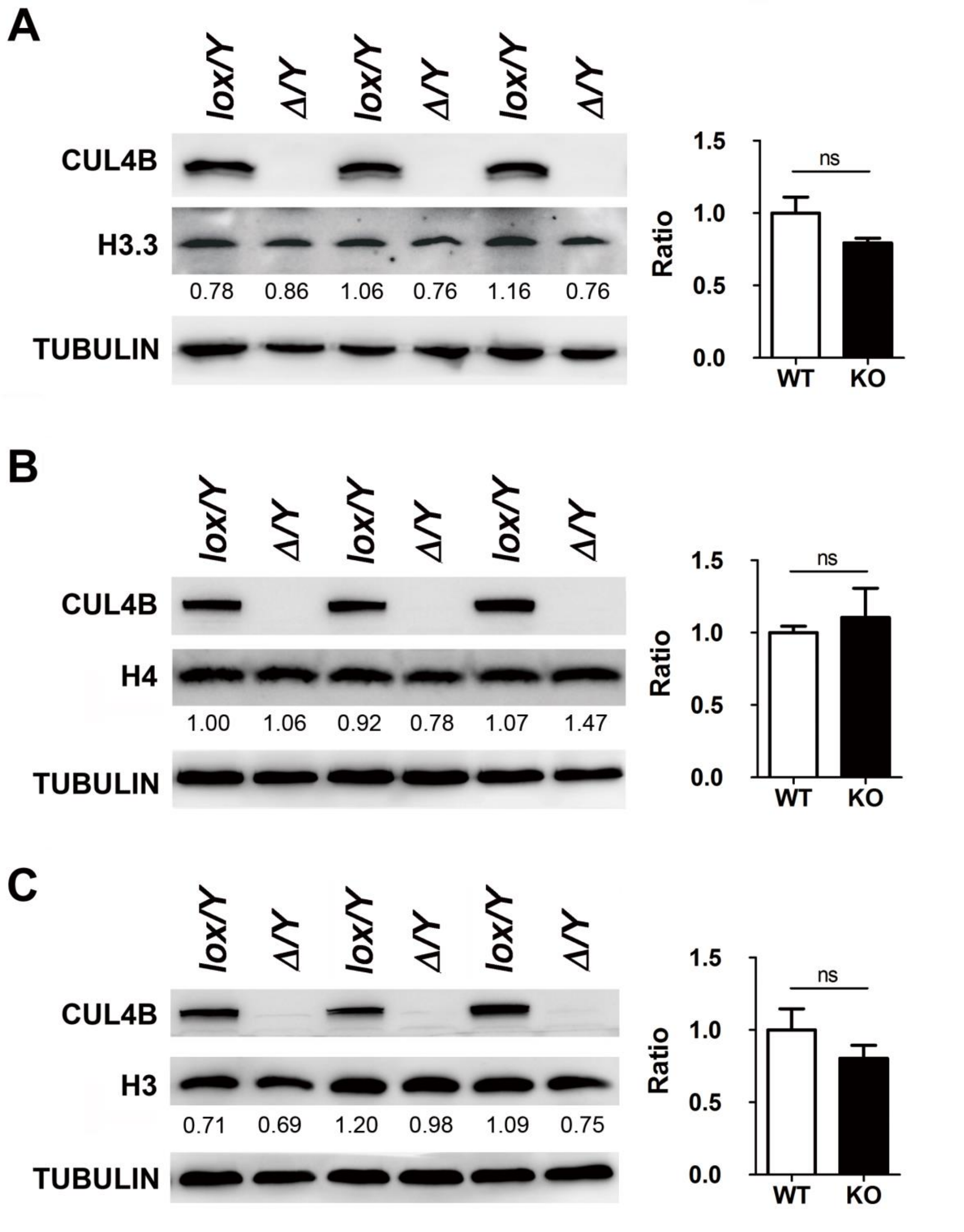
A
lox/Y
Δ/Y
CUL4B
H3.3
0.78 0.86 1.06 0.76 1.16 0.76
TUBULIN
Ratio
ns
WT
KO
B
H4
1.00 1.06 0.92 0.78 1.07 1.47
C
H3
0.71 0.69 1.20 0.98 1.09 0.75
